# A common neural signature between genetic and environmental risk for mental illness

**DOI:** 10.1038/s41398-025-03513-1

**Published:** 2025-08-21

**Authors:** Maria Vedechkina, Joni Holmes, Varun Warrier, Duncan E. Astle

**Affiliations:** 1 MRC Cognition and Brain Sciences Unit, University of Cambridge, Cambridge, UK; 2 School of Psychology, University of East Anglia, Norwich, UK; 3 Department of Psychiatry, University of Cambridge, Cambridge, UK

**Keywords:** Human behaviour, Diagnostic markers, Psychiatric disorders

## Abstract

Not everyone is equally likely to experience mental illness. What is the contribution of an individual’s genetic background and experiences of childhood adversity to that likelihood? And how do these risk factors interact at the level of the brain? This study explores these questions by investigating the relationship between genetic liability for mental illness, childhood adversity, and cortico-limbic connectivity in a large developmental sample drawn from the ABCD cohort (N = 6535). Canonical Correlation Analysis – a multivariate data-reduction technique – revealed two genetic dimensions of mental illness from the polygenic risk scores for ADHD, Anxiety, Depression, and Psychosis. The first dimension represented liability for broad psychopathology which was positively correlated with adversity. The second dimension represented neurodevelopmental-specific risk which negatively interacted with adversity, suggesting that neurodevelopmental symptoms may arise from unique combinations of genetic and environmental factors that differ from other symptom domains. Next, we investigated the cortico-limbic signature of adversity and genetic liability using Partial Least Squares. We found that the neural correlates of adversity broadly mirrored those of genetic liability, with adversity capturing most of the shared variance. These novel findings suggest that genetic and environmental risk *overlap* in the neural connections that underlie mental health symptomatology.

## Introduction

The prevalence of mental illness represents a significant global health challenge, with a myriad of factors contributing to its onset and progression [1]. Different combinations of interacting genetic, biological, and environmental constraints shape the neural substrates of mental health [2]. To better understand the etiology of common mental health conditions it is necessary to consider both genetic and environmental influences *in combination* and discern how they converge to produce risk-relevant neural and behavioural phenotypes [3–5].

The human genome interacts with the environmental factors to shape both physical and behavioural traits [6–9]. Genetic liability for mental illness is believed to manifest by altering the development of neural systems that play a role in symptom presentation, such as those involved in affective processing, fear learning, and social cognition [10–13]. The molecular embedding of adversity in stress-regulatory systems indicates that genetic and environmental risk may converge at the level of neurobiology [11, 13, 14] by altering stress-susceptible neural systems in similar ways [15–17]. Indeed, adversity exposed individuals often exhibit similar changes in brain structure and function to those typically observed in psychiatric patients, notably in the neural connections between limbic and cortical regions [18–22]. These neural differences may serve as partially heritable intermediate processes that mediate the link from genetic and environmental risk to more complex mental health symptamotology [23–25].

While the classification of psychiatric disorders has historically segmented mental health conditions into distinct categories, recent genetic studies have challenged the assumptions of disorder-specific risk by identifying a large number of *common* genetic variants with small effects, many of which predispose to *multiple* mental disorders [26–28]. Growing evidence supports a high degree of genetic correlation across different conditions [29, 30], indicating that the etiological and phenotypic boundaries between psychiatric disorders may not be as clear-cut as previously thought. Emerging evidence also suggests that correlations between childhood maltreatment and mental illness are driven partly by genetic factors [31]. However, much remains unknown about how these genetic risks manifest early in development. A key question motivating this work is whether genetic signals associated with psychiatric conditions in adulthood—such as schizophrenia, depression, or psychosis—are already detectable in childhood, and if so, how they relate to emerging symptoms and brain function. While many psychiatric disorders do not typically present until adolescence or later [32], their underlying genetic liability may influence preceding behavioural or neurobiological patterns. Investigating these early correlates can offer insight into how genetic and environmental risk factors unfold across development.

This study investigates how genetic and environmental risk factors converge to influence the neural substrates of mental illness in childhood, using data from a large cohort of 9–10-year-olds drawn from the Adolescent Brain and Cognitive Development (ABCD) study. We address three key questions: (1) To what extent do genetic and phenotypic risks overlap across psychiatric conditions? (2) How do genetic and environmental factors shape behavioural and neural profiles in childhood? (3) And finally, to what extent do these dimensions of risk converge at the level of brain connectivity?

First, we estimate polygenic risk scores (PRS) for four mental health phenotypes: ADHD, Anxiety, Depression, and Psychosis. We then use Canonical Correlation Analysis (CCA) to assess the degree of shared genetic liability across these mental health conditions, uncovering overlapping dimensions of genetic risk that transcend traditional phenotypic boundaries. Next, we examine how these dimensions of genetic liability relate to history of adversity. Finally, we use Partial Least Squares (PLS) to investigate the cortico-limbic signature of genetic and environment risk and identify neural markers of mental illness.

## Methods

### Participants

The Adolescent Brain Cognitive Development (ABCD) study is a longitudinal cohort that involves 21 data acquisition sites across the US and follows over 11,000 children aged 9–10 for 10 years into early adulthood [33, 34]. The study was designed to approximate the socio-demographic distribution of US children in this age group (Table S1). Participants were required to be aged 9–10 years at baseline for inclusion in the study. Those who lacked English language proficiency; suffered from severe sensory, intellectual, medical or neurological issues; or were unable to participate in MRI scanning were excluded. Recruitment details and data-collection procedures are described by Garavan et al. [33]. The current study uses data from participants with available genotype (n = 6535) and phenotype data as described below.

### Mental health measures

The parent-reported Child Behaviour Checklist (CBCL) is made up of 113 items rated on a three-point scale (not true; somewhat or sometimes true; very often or always true; Achenbach, 2011). These items are then summed into several subscales. This study uses the three DSM-oriented scales from the CBCL that align with clinical disorder definitions: Attention-Deficit Hyperactivity Disorder (ADHD), Depressive Disorder, and Anxiety Disorder. The scales have good inter-interviewer and test-retest reliability [35]. Raw scores from the baseline assessment (T1), which capture the number of total questions endorsed, were used (range: 0–20). Additionally, the Prodromal Psychosis Scale-Brief Child Version (PPS) was used to measure psychotic symptoms [36]. The original screening questionnaire, developed for adolescents and adults, was modified for use with children [37, 38]. The raw score from T1, based on the number of total questions endorsed, was used (range: 0–117).

### Early life adversity

A total of 24 questions were used to assess whether a child had been exposed to an adverse experience before the age of 10 (Table S2). Participants missing more than 15% of data on the adversity measures were removed from the analysis (n = 314) and the remaining missing answers were coded as ‘0’ (i.e., adversity not endorsed). These responses were coded as 0 because sensitivity analyses (reported in Supplement 1.2) revealed that either coding the missing responses as 1 (endorsing adversity) or using imputation resulted in estimates of adversity that were significantly higher than population prevalence estimates [39–41], indicating both approaches were heavily biased.

### MRI acquisition and preprocessing

ABCD standard imaging protocols for resting-state functional MRI (rsfMRI) including acquisition, processing, and quality assurance procedures have been described in detail elsewhere [42, 43]. Briefly, the preprocessing pipeline includes within- and between-scan head-motion correction, distortion corrections, removal of initial frames, normalisation, demeaning, regression, and temporal filtering [44, 45]. Average time courses for each region of interest (ROI) were calculated using FreeSurfer’s automated brain segmentation (aseg) and resampled to align with voxels from the fMRI data. Motion time courses are adjusted to account for signals linked to respiration (Hagler et al. [43]). This study uses imaging data from a subset of participants with 10 min of valid rsfMRI data below a framewise displacement threshold of 0.2 mm collected at T1 (n = 5995).

Using the processed rsfMRI data, parcellated time series were computed using a seed-based correlational approach [46]. Regions of interest (ROIs) were defined using the functional Gordon atlas template which comprises 352 ROIs (333 cortical) belonging to one of 13 networks (12 cortical and 1 subcortical) [47]. The functional connectivity between any two ROIs was estimated by calculating the lag-zero Pearson correlation coefficient of parcellated time series. This produced an ROI x ROI correlation matrix for each participant, which underwent an additional variance stabilization procedure using a Fischer z-transform [48].

### Polygenic risk score calculation

Saliva and blood samples were collected from participants as part of the ABCD biospecimen collection [49]. DNA extraction, basic biospecimen quality control, and genotyping using the Affymetrix Axiom Smokescreen Array were performed at the Rutgers University Cell and DNA Repository. Genotypes were called from the raw intensities using the Affymetrix Power Tools and the Affymetrix Best Practice Workflow in batch processing. The genomic coordinates were aligned with genome build hg19. To ensure data quality, cohort-level quality control (QC) procedures were implemented, which are described in detail elsewhere [9]. Samples missing more than 20% on genotype calls and variants with more than 10% missing rates were excluded during the initial QC. Samples with excessive relatedness were also removed. Individuals with sex discrepancies, such as those with ambiguous gender calls, were removed. Missing genotype data was inputted using the TOPMED reference panel with genome build GRCh38 and aligned with genome build hg19 [50].

After obtaining the minimally QCed genomic data, individuals from axiom plate 461, which was identified as problematic, were removed as recommended by the ABCD guidelines. Cryptic relatedness among samples was assessed, and one member of each first- and second-degree related pair was excluded to avoid confounding effects. Ambiguous SNPs and those with a missing allele were removed. PLINK 2.0 [51] was used for QC steps. Genetic variants with a call rate below 90% and minor allele frequency (MAF) less than 0.01 were removed to retain only common variants. Variants showing a significant departure from Hardy-Weinberg equilibrium (p < 1e-6) were filtered out to mitigate potential genotyping errors. Genetically related individuals with a first or second degree relative were removed. Pruning was performed to remove any highly correlated SNPs. Principal component analysis (PCA) was performed on the genotyping data to identify genetically inferred population ancestries to be used as covariates in the PRS calculation. Individuals with a heterozygosity rate of F>3sd from the population mean were removed. Duplicate SNPs and samples with a mislabelled sex chromosome were removed. A total of 6535 individuals passed all filters and QC steps.

Polygenic risk scores (PRS) were calculated for each of the four mental health phenotypes (see ‘Mental health measures’ above) using PRSice-2 software [52] based on a set of selected variants selected from the following case-control GWAS studies: (1) ADHD [53]; (2) Anxiety [54]; (3) Depression [55]; (4) Schizophrenia (for psychotic symptoms) [56]. Each of the studies defined cases based on clinical criteria, and controls were selected from individuals without the respective mental disorder. Variants reaching genome-wide significance (*p* < 5e-8) and those showing suggestive associations (*p* < 1e-5) were included in the PRS calculation[57]. PRSice-2 was run with the recommended settings, such as clumping and LD pruning thresholds, to control for linkage disequilibrium (LD) between selected variants [52]. Effect sizes and their standard errors for the selected variants were extracted from the GWAS summary statistics from each of the four studies. The PRS scores were calculated by summing the products of the effect sizes and the number of risk alleles carried by each individual at the selected variants for each of the four mental health phenotypes. The PRS calculation was also repeated separately for individuals from genetically inferred European (n = 5679) and non-European (n = 865) ancestries for sensitivity analyses. Descriptive statistics for study variables are shown in Table S40.

### Statistical analysis

#### Identifying shared dimensions of genetic risk

Canonical Correlation Analysis (CCA) was performed using the ‘CCA’ package in R [58] to explore the underlying relationships between the four PRS scores (Set A) and their respective phenotypes (Set B) and to identify patterns of correlation across the two datasets. We applied Canonical Correlation Analysis to identify latent overlapping factors between the set of polygenic risk scores and behavioural measures of mental health. This analysis yields paired linear combinations (canonical variates) of variables that are maximally correlated with each other [59]. Thus, CCA provides the ability to assess the extent of shared genetic variance across different mental health phenotypes. In contrast to other dimensionality-reduction techniques, CCA computes the components in the predictors and outcomes simultaneously such that they are maximally correlated with each other [60].

We calculated PRS component scores by adding up individual scores for each mental health dimension, weighted accordingly. Similarly, phenotype component scores were derived from a weighted sum of four mental health phenotypes. The first and second canonical correlations indicate the strength of associations between corresponding variate pairs, ranked by their correlation strength.

To determine the statistical significance of these correlations, we generated p-values using F-approximations of various test statistics. Canonical coefficients (or weights) were used to identify which variables had the strongest influence on each variate. We conducted a series of correlation analyses to assess how strongly each variable in our model was associated with component scores, providing a clearer interpretation than using canonical weights alone. As a sensitivity check for potential confounding due to genetic population differences, we performed supplementary CCA analyses. These included population substructure principal components (PCs) as covariates for the entire sample and specifically for individuals of European ancestry.

Finally, multiple linear regressions were conducted to assess how much variance in each mental health phenotype is explained by each of the PRS components, controlling age, sex, and the first 6 population substructure PCs. This allowed us to assess whether the PRS components explain more (or less) variance than disorder-specific PRS scores for each mental health phenotype. Because CCA variates are orthogonal to each other, meaning that each additional variate explains only the remaining variance not accounted for by previous components, all PRS component scores were entered jointly as predictors in the regression models. Confidence intervals for the multiple regressions were generated using bootstrapping with 1000 replicates.

#### Relationship between genetic liability for mental-ill health and adversity

To assess possible overlapping liability between genetic risk and adversity, multiple linear regressions were performed to examine the unique contribution of each PRS component score to variation in cumulative adversity, controlling for the respective phenotype component score, age, sex, and the first 6 population substructure principle components (PCs). Genetic population PCs were included as covariates to mitigate spurious associations between ancestry-based population-level stratification and outcomes of interest. For instance, studies have demonstrated correlations between variation in population structure and certain brain imaging features, as well as the prevalence of poverty in the US [61, 62]. Failure to control for genetically-defined population structure can therefore in spurious associations between brain measures, mental health, and adversity driven by uncorrected for population stratification within a given sample.

Next, multiple hierarchical linear regressions were conducted to assess whether PRS and adversity both independently predict mental health phenotypes and whether there is an interaction effect between genetic risk and the environment. Cumulative adversity scores were entered alongside each PRS component as predictors for the respective phenotype component, controlling age, sex, and the first 6 population substructure PCs. Interaction terms between PRS variates and adversity were added to the models as a second step. Change in R squared between the steps was used to assess differences in the overall model after adding the interaction term with corresponding significance tests.

#### Cortico-limbic signature of genetic and environmental risk

In our final analyses, we leveraged partial least squares (PLS) to assess how genetic and environmental risk covaries with cortico-limbic global and regional connectivity. PLS is a data-reduction technique well suited to capturing covariance and explaining complex relationships between a large set of noisy or multicollinear variables. It models the relationship between predictor and outcome variables by simultaneously projecting them to a new space to obtain a set of orthogonal latent variables that represent linear combinations of the original variables [63, 64]. We conducted two separate PLS analyses to model the covariance between adversity/PRS and (1) cortico-limbic global connectivity; and (2) cortico-limbic regional connectivity (regional-PLS).

In the first PLS, we modelled the four mental health PRSs and cumulative adversity as joint predictor variables (joint model), and connectivity between the limbic network and each of the 12 cortical networks as the outcome. We then repeated this using only the PRS scores (PRS-only model) and then only cumulative adversity (adversity-only model) as the predictor variables to assess unique associations between different dimensions of risk and cortico-limbic network connectivity. In our second PLS, we modelled the joint, PRS-only, and adversity-only predictors with connectivity between specific limbic regions (n = 19) and each of the cortical networks as the outcome to assess possible regional heterogeneity within the limbic network.

The *p*corr significance values for the PLS components were obtained by permuting the data 10,000 times and comparing the observed coefficients relative to their null distributions. Variable importance projection (VIP) was used to assess the relative importance of each variable in the model, with scores above 1 considered most influential in terms of their explanatory power. The stability of item loadings was assessed by averaging the mean squared error of prediction, R^2^, and Q^2^ across 10 cross-validation runs (nrepeat=10, folds=10), with a significance threshold of 0.01 for improvement in component error rate. To assess the stability of the model loadings, we employed a non-parametric bootstrapping approach with 1000 resampling iterations to obtain standard error, t-values, confidence intervals and p-values for each item. Each bootstrap sample was created by randomly resampling with replacement from the original dataset ensuring that the matrix structure for both predictor and response variables was preserved. Variables were scaled to unit variance prior to analysis. We regressed the latent variables obtained from each PLS model to obtain the latent component slope, with age, sex, and the 6 PCs added as covariates. Mental health phenotype scores were added as a second set of covariates. In the PRS-only model, adversity was added as a final covariate. In the adversity-only model, PRS scores were added as final covariates. For any significant regression results, we tested for possible mediating effects of the component response scores (i.e., latent scores based on cortico-limbic connectivity measures) on the association between the component predictor scores (i.e., latent scores based on PRS/adversity measures) and mental health. One thousand bootstrap samples were used to estimate the 95% confidence intervals for indirect effects using the bias-corrected percentile method proposed by Biesanz and colleagues (2010). Analyses were performed in R using the *MixOmics* [65], *caret* [66] and *mdatools* [67].

## Results

### Polygenic risk scores

**Fig. 1:**
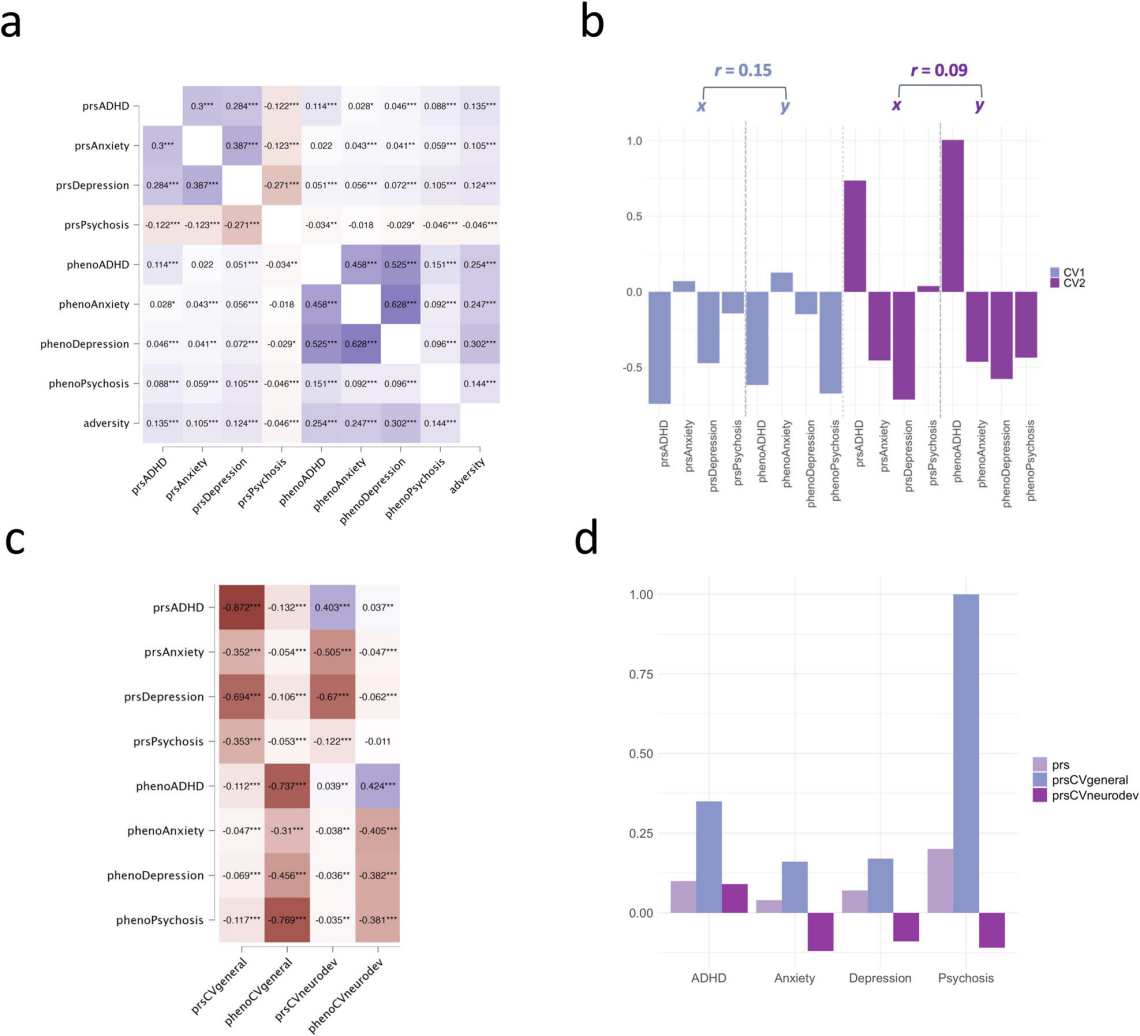
Canonical correlation analysis (CCA) for PRS scores and mental health phenotypes. **a** Correlations between genotype scores, their respective phenotypes, and adversity. **b** Scaled canonical correlation coefficients for each canonical variate. r= canonical correlation between each set of canonical components. **c** Canonical loadings showing the strength and significance of correlation of all variables with each canonical component. **d** Comparison of variance explained by disorder-specific PRS scores and the two PRS components. prsCVgeneral= PRS component scores from the first canonical variate representing general psychopathology. phenoCVgeneral= Phenotype component scores from the first canonical variate representing general psychopathology. prsCVneurodev= PRS component scores from the second canonical variate representing neurodevelopmental-specific variance. phenoCVneurodev= Phenotype component scores from the second canonical variate representing neurodevelopmental-specific variance. *p < 0.05, **p < 0.01, ***p < 0.001.

GWAS summary statistics from four mental health disorders (ADHD; Anxiety; Depression; Psychosis) were used to calculate PRS scores for four mental health phenotypes in the ABCD cohort for 6535 participants who passed all filters and QC steps (Figures S1–S6; Table S3). Hierarchical linear regressions assessing how much variance in each mental health phenotype is explained by each of the PRS scores, with age, sex, and the first 6 population substructure PCs as covariates are reported in Tables S4–S7. All four PRS scores were significantly correlated with their respective mental health phenotype and with adversity (Fig. 1a). The polygenic risk scores accounted for less than 1% of variance in mental health phenotypes, a pattern consistent with prior large-scale studies showing that PRS typically explain between 0.1–3% of variance in behavior, even in adult populations [68, 69].

### Shared dimensions of genetic risk

Canonical Correlation Analysis (CCA) was used to explore the underlying relationships between the four PRS scores and their respective phenotypes and identify possible genetic and phenotypic overlap across the four mental health conditions. The CCA yielded two significant canonical variates of modest strength with the following canonical correlation coefficients: Canonical Variate 1 (*r* = 0.15; Wilks’ λ = 0.97, F (16, 19950.12) = 13.23, *p* < 0.001); Canonical Variate 2 (*r* = 0.09; Wilks’ λ = 0.99, F (9, 15894.89) = 6.43, *p* < 0.001; Figure S7). The canonical loadings showing the corresponding strength and significance of correlations between disorder-specific mental health variables and each of the obtained genetic and phenotypic component scores are shown in Fig. 1 and Table S8. These loadings suggest that the first canonical variate represents a general risk for psychopathology across multiple conditions (henceforth referred to as ‘CVgeneral*’*), whereas the second represents unique neurodevelopmental-specific variance, characterised by positive loadings only from prsADHD (henceforth referred to as ‘CVneurodev*’*).

Multiple linear regressions demonstrating the amount variance in each mental health phenotype explained by the two PRS components (prsCVgeneral & prsCVneurodev), controlling for age, sex, and 6 genetically inferred population ancestry principal components (PCs) and comparing these results to the predictive value of the disorder-specific PRS scores are shown in Fig. 1 (also Tables S9–S12). The results did not qualitatively change when including population substructure PCs as covariates for the entire sample (Tables S34–S36) and for individuals with European ancestry only (Tables S37–S39). Overall, this suggests that genetic liability for different mental health conditions can be reduced to two independent transdiagnostic dimensions to predict variance in the respective behavioural phenotypes.

### Relationship between genetic liability for mental-ill health and adversity

**Fig. 2:**
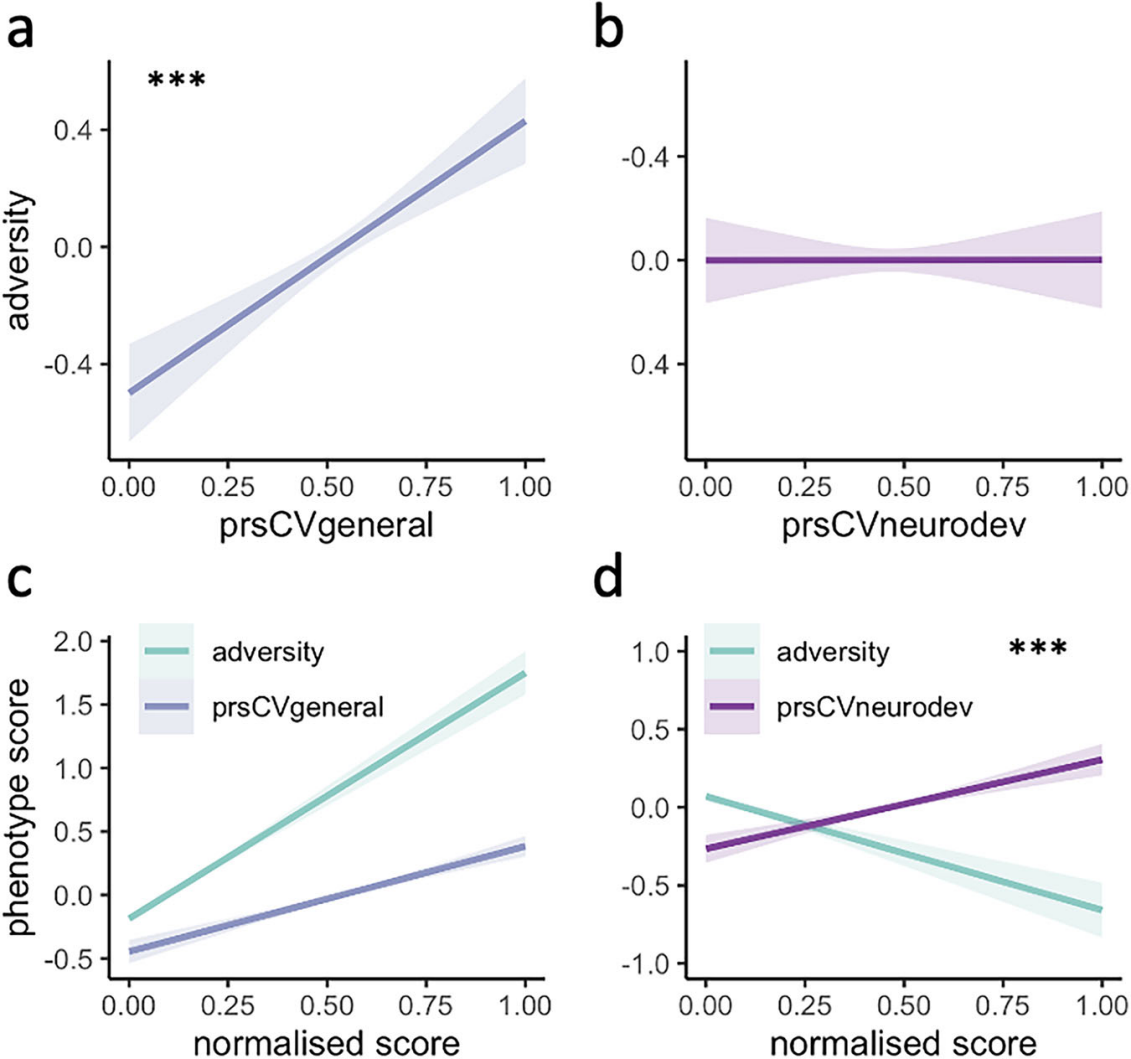
Associations between genetic liability dimensions and adversity. **a**, **b** Association between genetic liability components and adversity. prsCVgeneral= PRS component scores from the first canonical variate representing general psychopathology. prsCVneurodev= PRS component scores from the first canonical variate representing neurodevelopmental-specific variance. Regression models include age, sex, the first 6 population components (PCs), and the respective phenotype measures as covariates. **c**, **d** Prediction of phenotype components by PRS components, adversity, and their interaction. Regressions model the predictive value for each phenotype component separately, including age, sex, and the first 6 population components (PCs) as covariates. ***p < 0.001.

To investigate whether genetic liability for mental illness is related to adversity, multiple linear regressions were conducted with prsCVgeneral and prsCVneurodev as predictors, controlling for the respective CVpheno scores, age, sex, and the 6 PCs. prsCVgeneral (*b* = 0.089*, p* < 0.001), but not prsCVneurodev (*b* = 0.000, *p* = 0.99), was positively related to adversity (Tables S13–S14). prsCVgeneral (*b* = 0.116*, p* < 0.001) and adversity (*b* = 0.243*, p* < 0.001) both independently predicted phenoCVgeneral, but there was no interaction effect (Fig. 2a, c; full results in Table S19). In contrast, prsCVneurodev (*b* = 0.084*, p* < 0.001) and adversity (*b* = −0.098*, p* < 0.001) both independently predicted phenoCVneurodev— with similar strength but with opposing directions of effect— and there was also a significant interaction between prsCVneurodev and adversity (*b* = 0.043*, R*² *change* = 0.001, *p* = 0.006), indicating that increases in the effect of either variable attenuate the influence of the other (Fig. 2b, d; Table S20).

To test whether this pattern was specific to particular types of adversity, we repeated these analyses using four disaggregated adversity dimensions (household/community instability, physical/sexual abuse, parental neglect, financial difficulties) identified in prior work [70]. In the prsCVgeneral models, interaction effects were of similar magnitude across multiple adversity domains. In the prsCVneurodev model, the interaction effect was significant for the household/community instability factor, with a similar magnitude to the cumulative risk score (Tables S1–S2). This suggests that the composite adversity index offers a stable and efficient way to capture shared variance across adversity dimensions that is relevant for gene–environment interplay. The additional exploration of whether the effect holds across more specific adversity domains reveals some nuance to the prsCVneurodev model, with household/community instability being the strongest driver of that interaction.

### Cortico-limbic signature of genetic and environmental risk

#### Cortico-limbic global connectivity

The first PLS was used to model the covariance between genetic/environmental risk and connectivity between the entire limbic network (n = 1) and all cortical networks (n = 12). When using the four PRSs and adversity as joint predictors (joint model), a single significant component emerged (*p*perm < 0.001), explaining 33% of the variance in the predictors and 43% of the variance in the cortico-limbic network connections (Table S25). The correlation between the latent components was *b* = 0.03, SE = 0.009, *p* < 0.001 when controlling for age, sex and the 6PCs, and remained significant when mental health phenotype scores were added as covariates (*b* = 0.03, SE = 0.009, *p* < 0.001).

**Fig. 3:**
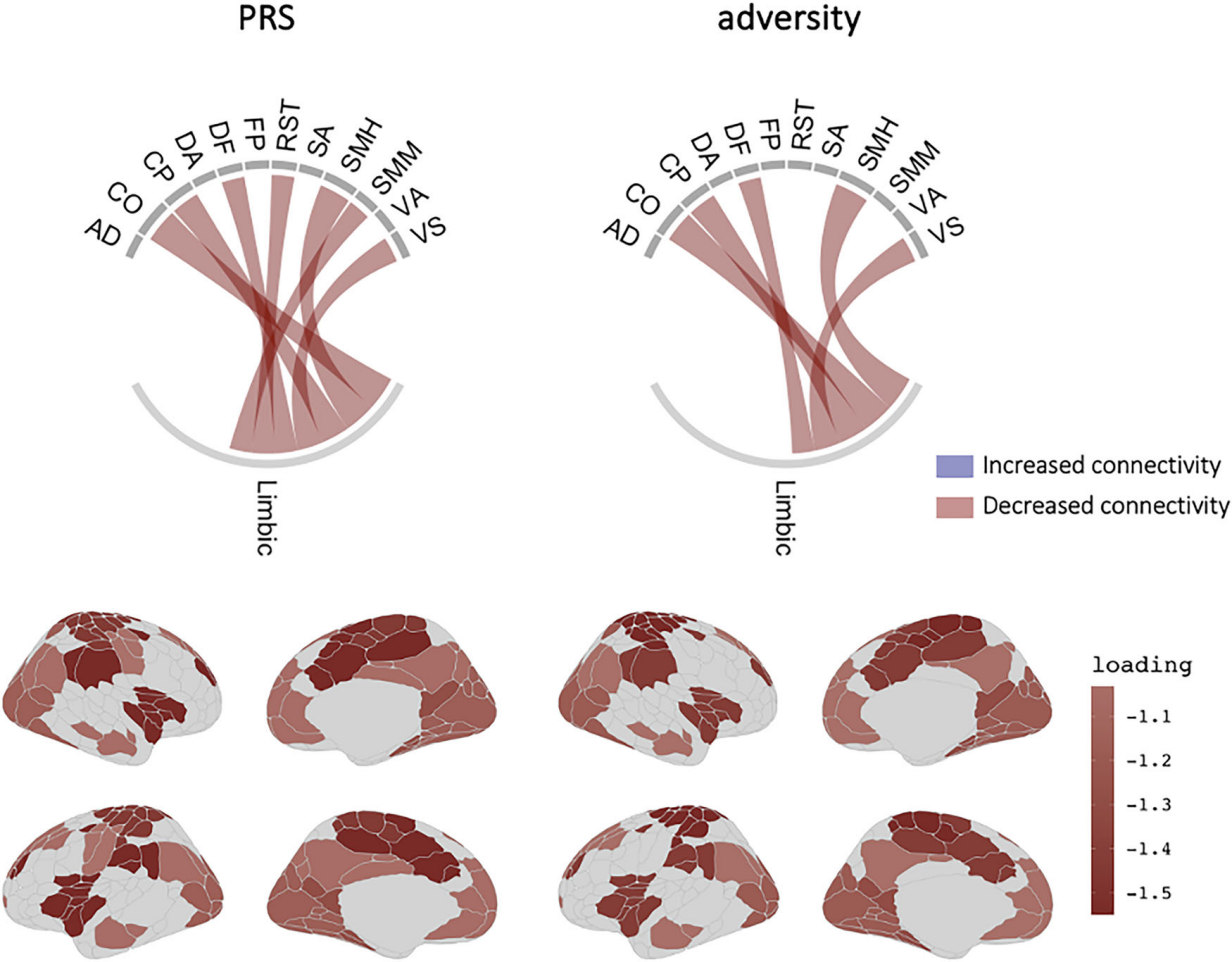
Cortico-limbic regional connectivity signature of genetic liability and adversity. PLS loadings of cortico-limbic network-level connectivity on the PRS-only model and adversity-only model. Only significant loadings with a VIP score above 1 shown in figure. AD auditory; CO cingulo-opercular; CP cingulo-parietal; DA dorsal attention; DF default; FP frontoparietal; RST retrosplenial temporal; SA salience; SMH sensorimotor hand; SMM sensorimotor mouth; VA ventral attention; VS visual.

When using only the four PRSs as predictor variables (PRS-only model), a single significant component emerged (*p*perm < 0.001), explaining 42% of the variance in the PRSs and 43% of the variance in the cortico-limbic network connections (Fig. 3; Table S26). The correlation between the latent components was *b* = 0.07, SE= 0.034, *p* = 0.04 when controlling for age, sex and the 6PCs, and remained significant when mental health phenotype scores were added as covariates (*b* = 0.07, SE = 0.034, *p* = 0.05). However, the CCA model became non-significant when adversity was added as a covariate (*b* = 0.05, SE = 0.034, *p* = 0.11).

When using only cumulative adversity as the predictor (adversity-only model), a single significant component emerged (*p*perm < 0.001), explaining 100% of the variance in adversity and 42% of the variance in the cortico-limbic network connections (Fig. 3; Table S27). The correlation between the latent components was *b* = 0.03, SE = 0.008, *p* < 0.001 when controlling for age, sex and the 6PCs, and remained significant when mental health phenotypes scores (*b* = 0.03, SE = 0.007, *p* < 0.001) and the PRS scores (*b* = 0.03, SE = 0.007, *p* < 0.001) were added as covariates. The cortico-limbic network component significantly mediated the association between the adversity component and symptoms of depression and psychosis (*p*’s = 0.032–0.033; Table S28).

Together, these analyses suggest that while adversity and the four genetic liability factors map onto common reductions in cortico-limbic connectivity at the *global* level, adversity captures much of this shared variance. The mediation analyses underscore the role of cortico-limbic circuitry as a potential neural pathway through which these risk factors may influence mental health symptomatology.

#### Cortico-limbic regional connectivity

A second PLS was used to model the covariance between genetic/environmental risk and connectivity between individual limbic regions (n = 19) and cortical networks (n = 12). When using the PRSs and adversity as joint predictors (joint model), a single significant component emerged (*p*perm < 0.001), explaining 33% of the variance in the predictors and 10% of the variance in the cortico-limbic regional connections. There were 70 significant regional connection loadings out of a total of 228 (Table S29). The correlation between the latent components was *b* = 0.022, SE = 0.04, *p* < 0.001 when controlling for age, sex and the 6PCs, and remained significant when mental health phenotype scores were added as covariates (*b* = 0.021, SE = 0.004, *p* < 0.001).

**Fig. 4:**
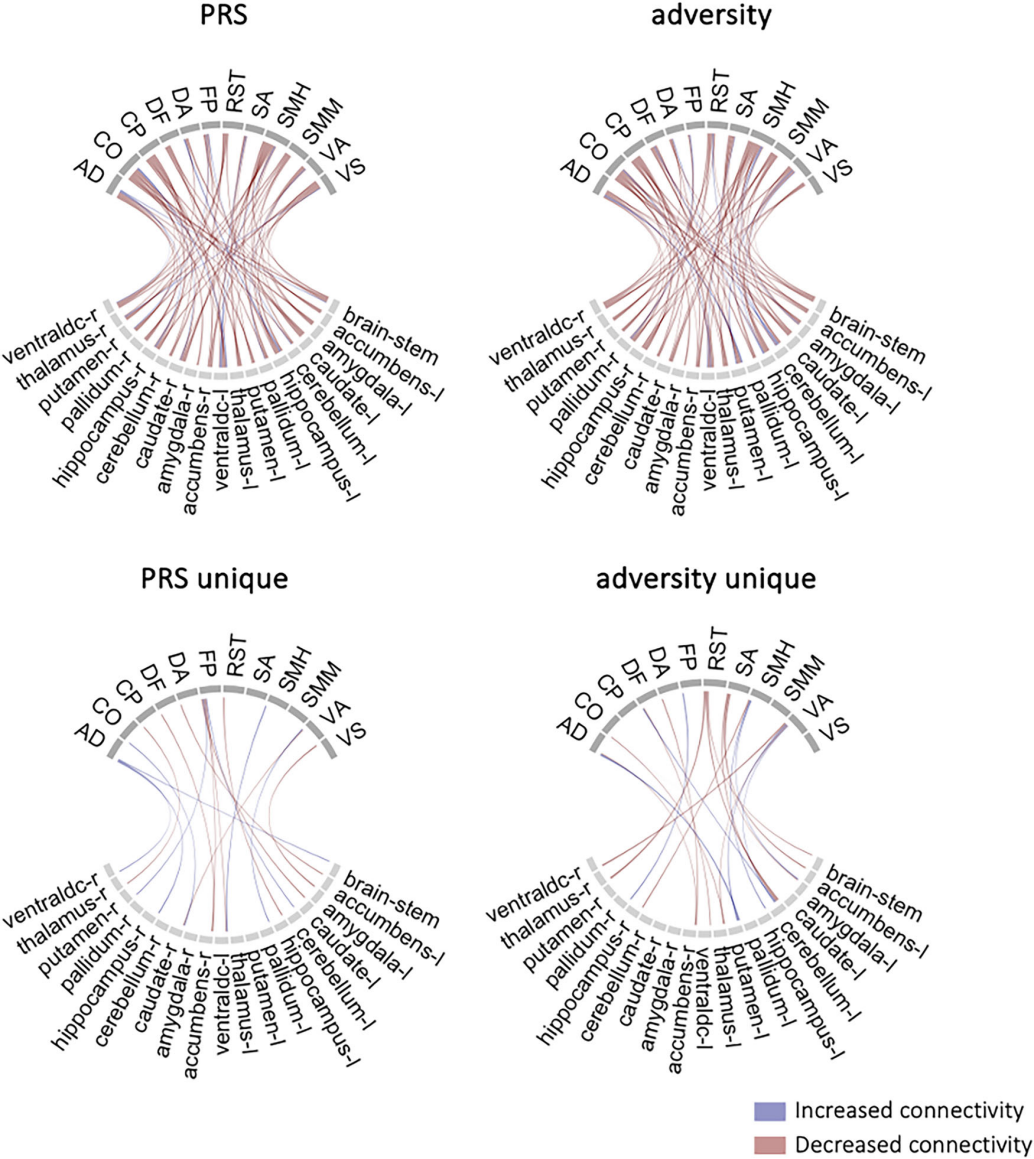
Cortico-limbic regional connectivity signature of genetic liability and adversity. The cortico-limbic regional connections identified by the partial least squares (PLS) model. PRS (polygenic risk): connections that significantly loaded onto the PLS model for genetic liability. Adversity: Connections that significantly loaded onto the PLS model for adversity. PRS unique: Connections that are uniquely associated with genetic liability. Adversity unique: Connections that are uniquely associated with adversity. Only significant loadings with a VIP score above 1 shown in figure. AD auditory; CO cingulo-opercular; CP cingulo-parietal; DA dorsal attention; DF default; FP frontoparietal; RST retrosplenial temporal; SA salience; SMH sensorimotor hand; SMM sensorimotor mouth; VA ventral attention; VS visual. The PRS and adversity models differed on 41 out of 147 significant loadings.

When using only the PRSs as predictor variables (PRS-only model), a single significant component emerged (*p*perm < 0.001), explaining 42% of the variance in the PRSs and 10% of the variance in the cortico-limbic regional connections. There were 73 significant connections out of a total of 228 (Fig. 4; Table S30). The correlation between the latent components was *b* = 0.015, SE = 0.005, *p* < 0.001 when controlling for age, sex and the 6PCs, and remained significant when mental health phenotype scores (*b* = 0.014, SE = 0.005 *p* = 0.001) and adversity (*b* = 0.013, SE = 0.005, *p* = 0.005) were added as covariates. The cortico-limbic regional component significantly mediated the association between the PRS component and symptoms of psychosis (*b* = −0.021, *p* = 0.038; Table S31).

When using only cumulative adversity as the predictor (adversity-only model), a single significant component emerged (*p*perm < 0.001), explaining 100% of the variance in the adversity and 10% of the variance in the cortico-limbic regional connections. There were 74 significant regional connections out of a total of 228 (Fig. 4; Table S32). The adversity-only model differed from the PRS-only model on 41 out of 147 significant loadings. Notably, the PRS-only model had greater loadings of limbic connectivity with the frontoparietal and visual networks, while the adversity-only model had greater loadings of limbic connectivity with the salience network. In contrast, there were few differences between the two models in terms of limbic connectivity with the cingulo-opercular, cingulo-parietal, and sensorimotor mouth networks. The correlation between the latent components was *b* = 0.023, SE = 0.004, *p* < 0.001 when controlling for age, sex and the 6PCs, and remained significant when mental health phenotypes scores (*b* = 0.023, SE = 0.004, *p* < 0.001) and the PRS scores (*b* = 0.021, SE = 0.004, *p* < 0.001) were added as covariates. The cortico-limbic regional component significantly mediated the association between the adversity component and symptoms of psychosis (*b* = −0.042, *p* = 0.006; Table S33).

Together, these analyses suggest that while adversity and the four genetic liability factors correspond to broadly similar regional differences in cortico-limbic connectivity, distinct patterns of connectivity associated with each dimension of risk become evident at the regional level of analysis. The mediation analyses underscore the role of cortico-limbic circuitry as a potential neural pathway through which both genetic and environmental risk factors may *independently* influence mental health symptomatology.

## Discussion

We investigated relationships between polygenic risk for mental illness, childhood adversity, and cortico-limbic connectivity in a large developmental sample drawn from the ABCD study and uncovered two novel findings. First, genetic liability for ADHD, anxiety, depression, and psychosis can be reduced to two pleotropic dimensions of risk, each of which uniquely interact with exposure to adversity. Second, genetic and environmental risk factors overlap in their neural correlates. This shared neural signature indicates a gene-environment correlation at the level of the brain, with substantial implications for future research on the neural correlates and etiology of mental illness.

Our findings add to a growing body of evidence that traditional diagnostic boundaries may not map onto distinct neurobiological processes [27, 71, 72]. Given the common genetic origins and phenotypic overlap between disorder categories [28, 29, 73], existing disorder-specific GWAS studies may require re-evaluation. Furthermore, our study demonstrates significant correlations between genetic liability for psychopathology and environmental adversity in this sample [31, 74, 75]. Specifically, we found that genetic liability for general psychopathology was positively associated with adversity, suggesting an additive effect, whereby both factors appear to exert independent, cumulative influence on symptom severity [76]. In contrast, the association between adversity and neurodevelopmental symptoms was attenuated in individuals with higher genetic risk, suggesting a compensatory or interaction effect whereby one source of risk (genetic or environmental) reduces the influence of the other on symptom presentation. This points to the likelihood that neurodevelopmental symptoms may arise from combinations of genetic and environmental factors unique from other symptom domains. This could reflect a degree of etiological independence: genetic factors underlying ADHD/neurodevelopmental problems may influence brain development regardless of the child’s environmental adversity level. However, it should be noted that the adversity composite included items like parental psychiatric history and family psychosocial stressors. As such, higher polygenic risk for general psychopathology is more likely to co-occur with certain adversity indicators due to shared familial risk factors, potentially inflating the observed correlation for general liability.

Our study also highlights that environmental and genetic risk largely overlap in the neural connections that underlie mental health symptom variance. Both adversity and genetic liability were associated with reduced functional connectivity between the limbic and the cingulo-opercular, cingulo-parietal, visual, default, and sensorimotor networks. This global pattern of altered connectivity indicates that *widespread* disruptions in cortico-limbic connectivity are a core feature of transdiagnostic symptomatology [77–80]. In other words, pleotropic risk genes for multiple disorders *and* environmental risk factors increase susceptibility to a variety of clinically-distinct psychiatric conditions though non-specific changes in cortico-limbic connections by altering stress-susceptible systems in similar ways [15–17, 81].

However, our regional analyses also revealed notable heterogeneity *within* the limbic network, with 41 out of 147 cortico-limbic connections showing unique associations with genetic risk. The strongest effects emerged in connections with the frontoparietal and visual networks, suggesting that these circuits may be particularly sensitive to polygenic influences [82, 83]. These modest and spatially distributed PRS–brain associations are in line with findings from large-scale imaging genetics studies, which consistently report that individual genetic variants account for less than 0.1% of variance in brain structure or function, and that polygenic effects are typically diffuse rather than localized [83, 84]. Thus, although the observed genetic effects were weaker than those for adversity, their distribution and magnitude are consistent with current expectations for polygenic contributions to brain connectivity. Importantly, as GWAS sample sizes grow, PRS effect sizes tend to increase not by implicating entirely new biological mechanisms, but by capturing more variants within the same underlying molecular and cellular pathways [85, 86]. This suggests that even modest early signals may reflect biologically meaningful risk processes that become more apparent over time.

The finding that adversity accounted for the entirety of the genetic variance at the global level suggests that many of the previously identified neural markers of psychopathology may be capturing adversity-related variance, and vice-versa. In other words, the neural features stemming from early adversity are likely conflated with those that predispose individuals to mental illness in the existing literature [19]. Although many recent studies incorporate controls for co-occurring psychiatric symptoms, this has not been a consistent feature of earlier research on adversity-related neural outcomes [12, 19]. Even in contemporary work, approaches vary widely, with some studies adjusting for broad symptom indices, others focusing only on select diagnoses, and many omitting psychiatric covariates altogether [85, 87–89]. This methodological inconsistency complicates efforts to isolate the unique neurobiological effects of adversity and genetic risk and should be carefully considered when interpreting findings across the existing literature.

Efforts to identify genetically driven neural endophenotypes of mental illness [90] would do well to recognise this as a key methodological challenge for the field. Future research would benefit from stratifying mental health conditions based not only on clinical symptoms and neurobiological features, but also on genetic *and* environmental risk factors. Such an approach could pave the way towards precise diagnoses, endophenotypes, and tailored treatments [91–93] that are more effective for certain individuals and populations [94–96].

The strengths of this study lie in its application of diverse methods to capture multicollinear associations between genetic liability, environmental risk, and functional brain connectivity. However, some limitations must also be acknowledged. First, the study sample is representative of the US context and was restricted to children aged 9–10, limiting the generalisability of the findings to other demographic groups and age ranges. Future longitudinal research should endeavour to replicate these findings in diverse demographic groups across development. Second, while the behavioural expression of mental health difficulties may vary at different developmental time points [97, 98], this study only considered four mental health conditions due to data availability constraints. Third, while the use of dimensional symptom scales circumvents some shortcomings inherent to clinical diagnostic categories [99], it is worth noting that only a small fraction of the sample met the threshold for a clinical diagnosis. This is likely because our sample was composed of children aged 9–10, prior to the typical onset of psychiatric disorders like schizophrenia and major depression [32]. Consequently, polygenic risk for these conditions may not yet be phenotypically expressed in behavior or neurobiology [100]. Indeed, the stronger association of adversity with functional connectivity in our study may reflect the relatively low behavioural symptom variance explained by the PRS scores. Furthermore, connectivity differences related to psychopathology risk may still be emerging at age 9–10, given the ongoing maturation of cortico-limbic networks [101, 102]. While our findings suggest shared genetic and neural patterns across multiple symptom domains, we emphasize that these associations are modest in magnitude and developmentally constrained. At age 9–10, many psychiatric conditions have not yet fully emerged, and the neural signatures of risk may still be evolving. Accordingly, the associations we observed likely reflect early, subclinical markers of genetic liability, rather than predictors of imminent psychopathology. Nonetheless, these findings contribute to a growing transdiagnostic literature by demonstrating that polygenic and environmental risk factors can converge on shared neurobiological pathways prior to clinical onset [69, 103]. Longitudinal follow-up of this cohort will be necessary to determine whether these early associations strengthen or change over time [68]. It is also necessary to validate these findings using genetic methods better suited to capturing shared genetic signals across multiple conditions [104].

In line with established cumulative risk frameworks [105], we used a composite adversity score to capture the overall burden of early life adversity. Aggregating across diverse exposures helps reduce measurement error and reflects the fact that children rarely experience isolated adverse events; rather, multiple adversities often co-occur and interact developmentally [106]. While this approach maximizes power and supports dimensional modelling across neurobehavioral domains, we acknowledge that collapsing adversity into a single index may potentially mask distinct effects of specific adversity types. However, our supplemental analyses suggest that disaggregation did not reveal stronger, but did suggest that household/community instability to be the strongest driver with regard to the prsCVneurodev interaction. In prior work using the same ABCD dataset [70], we also found that attempts to disaggregate adversity into threat and deprivation dimensions were limited by multicollinearity and did not yield clearly distinct neural correlates. Nonetheless, future work could benefit from more refined measurement models in samples where these constructs are psychometrically separable, to explore potential specificity of effects.

In conclusion, this study demonstrates that comparable neural differences can emerge from both endogenous (genetic) and exogenous (environmental) risk factors. Our findings add to the growing evidence of overlapping etiological influences across symptom domains, even in a pediatric sample. Future longitudinal research will be critical to determine the clinical significance of these early patterns and to further elucidate how genetic and environmental factors jointly shape the neural substrates of mental illness over the course of development.

## Supplementary information

Supplementary Materials

## Data Availability

The datasets analysed during the current study are available in the NIMH Data Archive (NDA) repository, 10.15154/1523041.

## Code availability

The underlying code for this study is not publicly available but may be made available to qualified researchers on reasonable request from the corresponding author.
