# Supplementary material for: A common neural signature between genetic and environmental risk for mental illness
Source: Transl Psychiatry. 2025 Aug 21;15:305. doi: 10.1038/s41398-025-03513-1 (PMC12371083; doi:10.1038/s41398-025-03513-1)
Supplement: Supplementary file 1 — Supplementary Materials [file 41398_2025_3513_MOESM1_ESM.pdf]

1. METHODS SUPPLEMENT

Table S1. Demographics for all ABCD participants at baseline

N	11,876
Age mean *(SD)*	9.9 *(0.6)*
% Female	47.8%
Race/Ethnicity	
% White	52.2%
% African American	15.1%
% Hispanic	3.9%
% Asian	2.2%
% Other/Multi-racial	25.9%
Household characteristics	
% Married caregivers	65.5%
% College-level education	59.4%
Household income	
< $25k	17.2%
$25k - $49.99k	22.4%
$50k -$74.99k	14.5%
$75k -$99.99k	30.5%
$100k +	15.4%

Notes. Age reported in years. College education reported if one or more caregivers has a college-level degree.

Table S2. Adversity questions

Description	ABCD variable name	Timescale	% missing
parent alcohol problem	famhx_ss_momdad_alc_p	Lifetime (age 0-9)	3.77
parent drug use problem	famhx_ss_momdad_dg_p	Lifetime (age 0-9)	5.45
parent trouble with job/fights/police	famhx_ss_momdad_trb_p	Lifetime (age 0-9)	3.05
community shooting or stabbing	ksads_ptsd_raw_760_p	Lifetime (age 0-9)	2.41
shot, stabbed, beaten by non-family	ksads_ptsd_raw_761_p	Lifetime (age 0-9)	2.41

shot, stabbed, beaten by caregiver	ksads_ptsd_raw_762_p	Lifetime (age 0-9)	2.41
severely beaten by caregiver	ksads_ptsd_raw_763_p	Lifetime (age 0-9)	2.41
death threat by non-family	ksads_ptsd_raw_764_p	Lifetime (age 0-9)	2.41
death threat by family	ksads_ptsd_raw_765_p	Lifetime (age 0-9)	2.41
interparental violence	ksads_ptsd_raw_766_p	Lifetime (age 0-9)	2.41
sexual abuse by caregiver	ksads_ptsd_raw_767_p	Lifetime (age 0-9)	2.41
sexual abuse by non-family	ksads_ptsd_raw_768_p	Lifetime (age 0-9)	2.41
sexual abuse by peer	ksads_ptsd_raw_769_p	Lifetime (age 0-9)	2.41
unsafe community	nsc_p_ss_mean_3_items	Lifetime (age 0-9)	0.07
poor parental supervision	pmq_y_ss_mean	Lifetime (age 0-9)	0.18
low caregiver acceptance	crpbi_y_ss_parent	Lifetime (age 0-9)	0.29
financial difficulties: food	demo_fam_exp1_v2	Past 12 months	0.64
financial difficulties: phone service	demo_fam_exp2_v2	Past 12 months	0.39
financial difficulties: rent payment	demo_fam_exp3_v2	Past 12 months	0.51
financial difficulties: eviction	demo_fam_exp4_v2	Past 12 months	0.28
financial difficulties: gas and electric	demo_fam_exp5_v2	Past 12 months	0.37
financial difficulties: medical care	demo_fam_exp6_v2	Past 12 months	0.34
financial difficulties: dentist	demo_fam_exp7_v2	Past 12 months	0.41
sudden death of loved one	ksads_ptsd_raw_770_p	Lifetime (age 0-9)	2.41

Notes. Adversity items taken at baseline assessment (T1). Parental report used. Missingness represents the percentage of participants with missing data on a given question.

1.2 Early life adversity

Missing adversity data was coded as "0" because sensitivity analyses revealed that either coding it as 1 or imputing it resulted in an overestimation of adversity in the sample relative to population prevalence estimates (Finkelhor et al., 2005; McLaughlin et al., 2012; Struck et al., 2020). In the first sensitivity analysis, we coded missing data as 1 instead of 0. This resulted in an additional 524 participants with adversity exposures, and an ELA group representing 27% of the total sample. This was much higher than population prevalence estimates would suggest, meaning this approach was heavily biased unlikely representative of real-world data.

Next, we used a multiple imputation package for mixed-type data, missForest in R (Stekhoven et al., 2012) to impute the missing values. Imputing missing values resulted in an additional 512 participants with adversity exposures (1251 in; 26.5% of the total sample), again much higher than population prevalence estimates (Finkelhor et al., 2005; McLaughlin et al.,

2012; Struck et al., 2020). Imputation algorithms are heavily biased towards rare cases with binary data (e.g., exposures to ELA). This means adversity is likely over-estimated, explaining why using this method resulted in an unusually high number of children classified as having experienced adversity relative to population prevalence estimates (Finkelhor et al., 2005; McLaughlin et al., 2012; Struck et al., 2020). There were other reasons for not using imputation. First, it would have increased the standard error, which would have been problematic for our subsequent analyses. Second, the data were not missing at random. We tested for associations between missingness and several key cognitive and demographic variables. Missingness was associated interview ethnicity (p<.001); parental education (p<.001); parental income (p<.001) and 3 out of 5 measures of cognition that were tested (ps= .001-.05). The missing at random assumption was therefore not plausible, meaning that imputation would be heavily biased. Although imputation is beneficial in some cases, it must be balanced against the possible risks of inducing bias and overfitting, particularly in the case of non-normally distributed binary data (Sterne et al., 2009). While some procedures can handle non-normally distributed data better than others (Van Buuren et al., 1999), it is an ongoing area of development (Horton et al., 2007; Bernaards et al, 2007) that currently has no well-defined solution (Lee & Carlin, 2016; Sullivan et al., 2017). For these reasons, we decided imputation was not appropriate for our data.

2. RESULTS SUPPLEMENT

Figure S1. Results for PRS ADHD

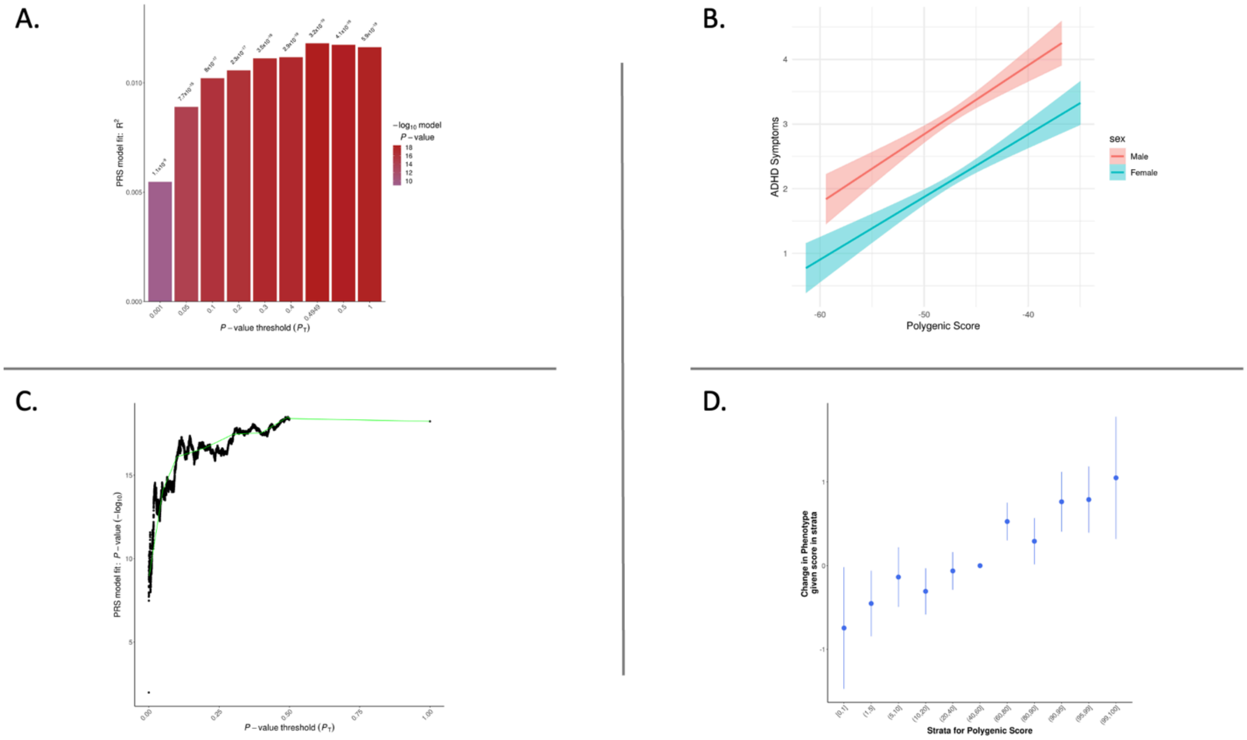

Notes. (A) Bar plot displaying the model fit of the PRS at different p-value thresholds; (B) PRS best fit results; (C) High resolution plot showing model fit at all p-value thresholds. The green line connects points showing the model fit at broad p-value thresholds used in the corresponding bar plot; (D) Strata plot providing an illustration of the effect of increased PRS on predicted phenotype using an uneven distribution of deciles.

Figure S2. Results for PRS Anxiety

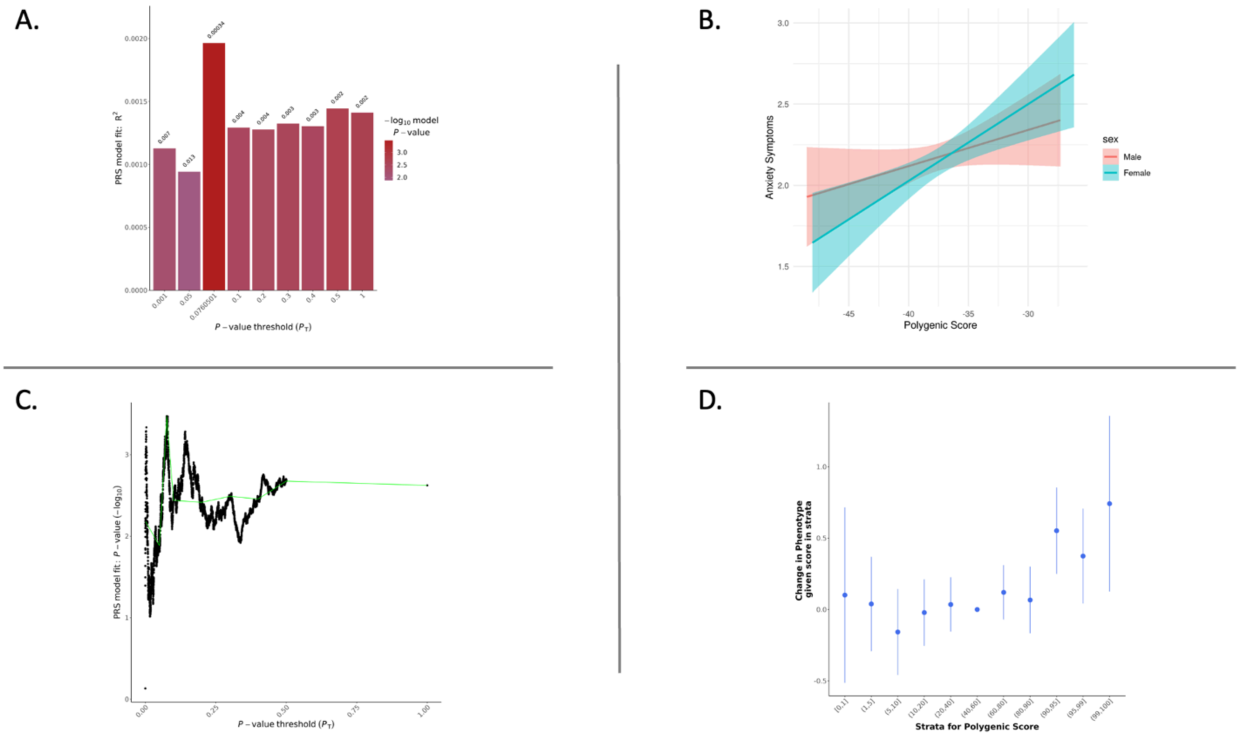

Notes. (A) Bar plot displaying the model fit of the PRS at different p-value thresholds; (B) PRS best fit results; (C) High resolution plot showing model fit at all p-value thresholds. The green line connects points showing the model fit at broad p-value thresholds used in the corresponding bar plot; (D) Strata plot providing an illustration of the effect of increased PRS on predicted phenotype using an uneven distribution of deciles.

Figure S3. Results for PRS Depression

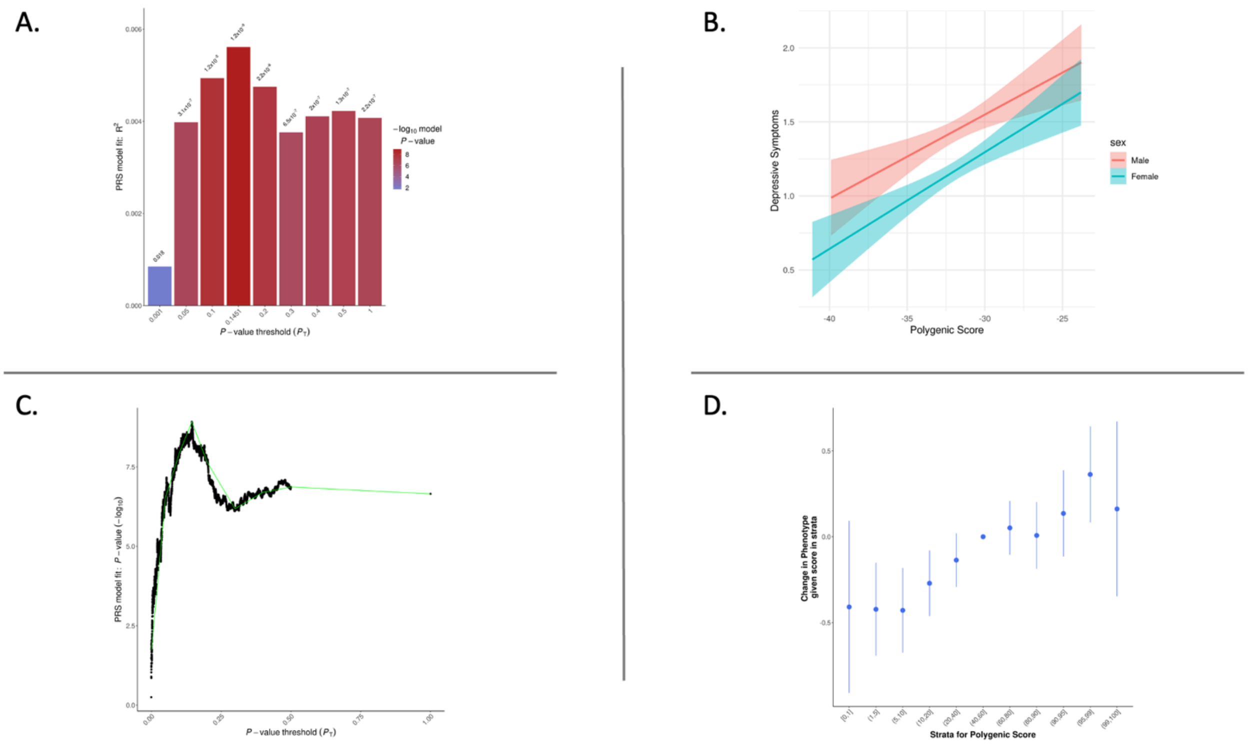

Notes. (A) Bar plot displaying the model fit of the PRS at different p-value thresholds; (B) PRS best fit results; (C) High resolution plot showing model fit at all p-value thresholds. The green line connects points showing the model fit at broad p-value thresholds used in the corresponding bar plot; (D) Strata plot providing an illustration of the effect of increased PRS on predicted phenotype using an uneven distribution of deciles.

Figure S4. Results for PRS Psychosis

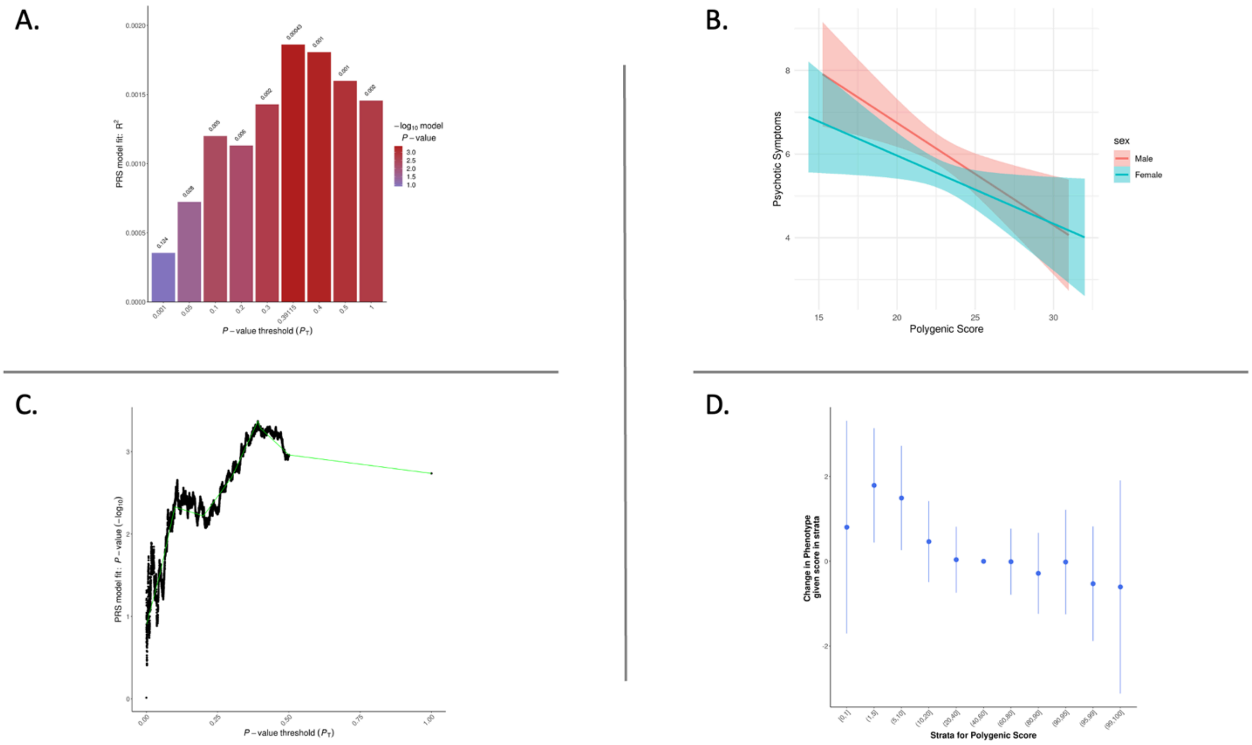

Notes. (A) Bar plot displaying the model fit of the PRS at different p-value thresholds; (B) PRS best fit results; (C) High resolution plot showing model fit at all p-value thresholds. The green line connects points showing the model fit at broad p-value thresholds used in the corresponding bar plot; (D) Strata plot providing an illustration of the effect of increased PRS on predicted phenotype using an uneven distribution of deciles.

Table S3. PRS best fit results computed separately for participants of European and non-European descent.

Phenotype	Base GWAS	Thresh	PRSR2	FullR2	NullR2	Coef	Std Err	P	SNPs
European descent									
ADHD	Demontis 2023	1.000	0.014	0.042	0.029	0.102	0.012	0.0000	79009
Anxiety	Meier 2019	0.079	0.003	0.005	0.003	0.042	0.011	0.0002	9240
Depression	Wray 2018	0.093	0.007	0.014	0.007	0.086	0.014	0.0000	131669
Psychosis	Richards 2022	0.391	0.001	0.014	0.013	-0.158	0.059	0.0074	48515
Non-European descent									
ADHD	Demontis 2023	0.000	0.011	0.071	0.060	0.775	0.211	0.0003	183
Anxiety	Meier 2019	0.000	0.007	0.023	0.016	0.427	0.148	0.0040	97
Depression	Wray 2018	0.000	0.004	0.016	0.011	-4.074	1.786	0.0227	1
Psychosis	Richards 2022	0.109	0.008	0.026	0.018	-0.624	0.206	0.0025	19598

Notes. Thresh= best p-value threshold. PRS R2= Variance explained by the PRS. Full R2= Variance explained by the full model (including covariates age, sex, and 6 PCs). Null R2= Variance explained by the covariates. Coef, Std Err and P= Regression coefficient, standard error, and significance for the model. SNPs= Number of SNPs included in the model.

Figure S5. Distribution of the PRS scores within the entire sample

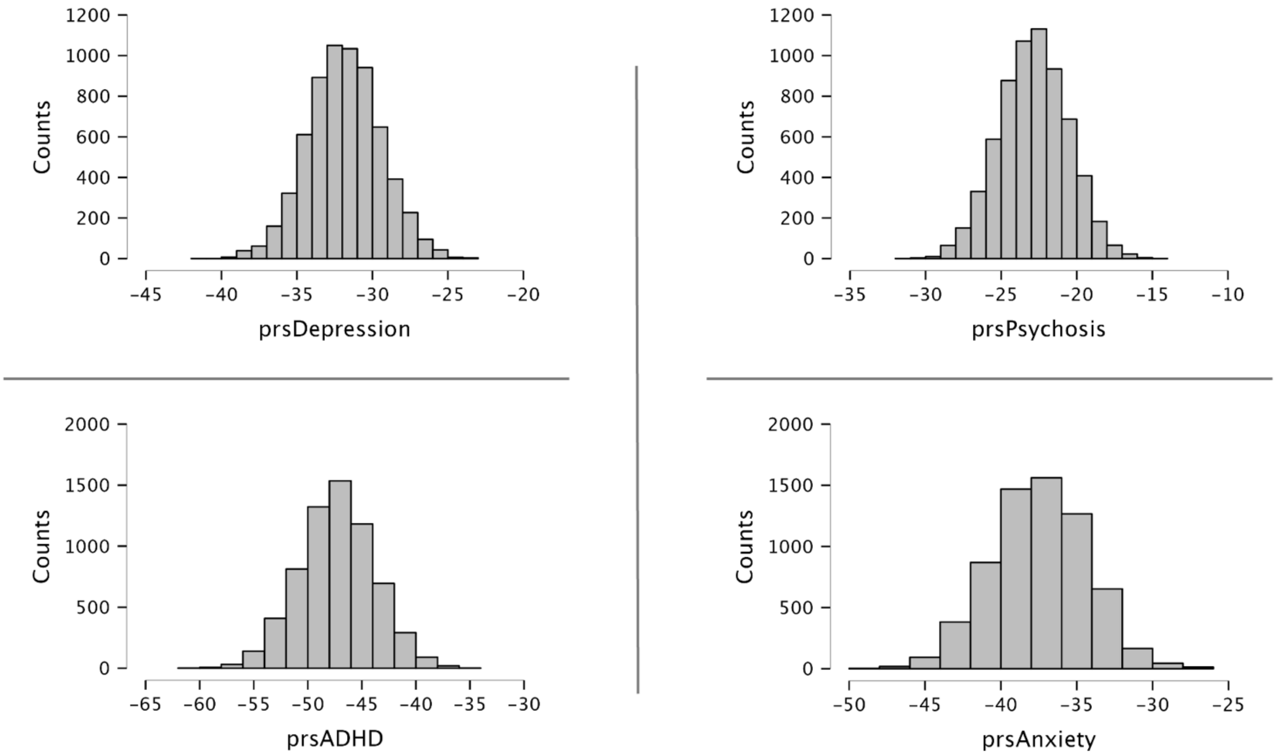

Table S4. Hierarchical linear regression for variance explained in ADHD symptoms by ADHD PRS

Model		Coef	Std Err	Std Coef	t	p
H_0	*(Intercept)*	5.05	0.59		8.58	0.000
	age	-0.02	0.01	-0.04	-3.29	0.001
	sex	-0.99	0.07		-13.53	0.000
	PC1	-12.57	2.95	-0.05	-4.26	0.000
	PC2	9.95	2.95	0.04	3.37	0.001
	PC3	-3.60	2.95	-0.02	-1.22	0.222
	PC4	-8.73	2.95	-0.04	-2.96	0.003
	PC5	-5.20	2.94	-0.02	-1.77	0.078
	PC6	-0.14	2.95	0.00	-0.05	0.962
H_1	*(Intercept)*	9.79	0.79		12.43	0.000
	prsADHD	0.10	0.01	0.12	8.99	0.000
	age	-0.02	0.01	-0.04	-3.34	0.001
	sex	-1.00	0.07		-13.76	0.000
	PC1	-4.39	3.07	-0.02	-1.43	0.153
	PC2	9.81	2.93	0.04	3.35	0.001
	PC3	-6.00	2.94	-0.03	-2.04	0.042
	PC4	-7.37	2.93	-0.03	-2.51	0.012
	PC5	-7.36	2.94	-0.03	-2.51	0.012
	PC6	-0.63	2.93	0.00	-0.22	0.830

Model	R^2	Adjusted R^2	R^2 Change	df1	df2	p
H_0	0.035	0.034	0.035	8	6526	0.000
H_1	0.047	0.045	0.012	1	6525	0.000

Notes. Null model includes age, sex, PC1, PC2, PC3, PC4, PC5, PC6.

Table S5. Hierarchical linear regression for variance explained in anxiety symptoms by Anxiety PRS

Model		Coef	Std Err	Std Coef	t	p
H_0	*(Intercept)*	2.76	0.50		5.58	0.000
	age	-0.01	0.00	-0.02	-1.19	0.234
	sex	-0.03	0.06		-0.41	0.680
	PC1	-1.67	2.48	-0.01	-0.67	0.501
	PC2	1.58	2.48	0.01	0.64	0.523
	PC3	-5.55	2.48	-0.03	-2.24	0.025
	PC4	-0.18	2.48	0.00	-0.07	0.943
	PC5	-4.27	2.48	-0.02	-1.73	0.084
	PC6	-4.02	2.48	-0.02	-1.62	0.105
H_1	*(Intercept)*	4.17	0.63		6.62	0.000
	prsAnxiety	0.04	0.01	0.05	3.60	0.000
	sex	-0.03	0.06		-0.45	0.650
	age	-0.01	0.00	-0.02	-1.24	0.217
	PC1	0.62	2.56	0.00	0.24	0.809
	PC2	2.78	2.50	0.01	1.11	0.266
	PC3	-5.91	2.48	-0.03	-2.39	0.017
	PC4	-1.06	2.49	-0.01	-0.42	0.671
	PC5	-4.17	2.47	-0.02	-1.68	0.092
	PC6	-3.91	2.48	-0.02	-1.58	0.114
**Model**	**R^2**	**Adjusted R^2**	**R^2 Change**	**df1**	**df2**	**p**
H_0	0.002	0.001	0.002	8	6526	0.103
H_1	0.004	0.003	0.002	1	6525	0.000

Notes. Null model includes age, sex, PC1, PC2, PC3, PC4, PC5, PC6.

Table S6. Hierarchical linear regression for variance explained in depression symptoms by Depression PRS

Model		Coef	Std Err	Std Coef	t	p
H_0	*(Intercept)*	0.91	0.41		2.25	0.025
	age	0.00	0.00	0.02	1.32	0.186
	sex	-0.27	0.05		-5.34	0.000
	PC1	-4.58	2.03	-0.03	-2.26	0.024
	PC2	3.09	2.03	0.02	1.52	0.128
	PC3	-3.97	2.03	-0.02	-1.96	0.050
	PC4	-0.23	2.03	0.00	-0.11	0.911
	PC5	-3.92	2.03	-0.02	-1.94	0.053
	PC6	-1.80	2.03	-0.01	-0.89	0.374
H_1	*(Intercept)*	3.25	0.56		5.83	0.000
	prsDepression	0.07	0.01	0.09	6.09	0.000
	age	0.00	0.00	0.02	1.20	0.230
	sex	-0.27	0.05		-5.32	0.000
	PC1	1.28	2.24	0.01	0.57	0.569
	PC2	6.01	2.08	0.04	2.89	0.004
	PC3	-4.39	2.02	-0.03	-2.17	0.030
	PC4	-1.55	2.03	-0.01	-0.76	0.446
	PC5	-4.16	2.02	-0.03	-2.06	0.039
	PC6	-2.00	2.02	-0.01	-0.99	0.322

Model	R^2	Adjusted R^2	R^2 Change	df1	df2	p
H_0	0.007	0.006	0.007	8	6526	0.000
H_1	0.013	0.011	0.006	1	6525	0.000

Notes. Null model includes age, sex, PC1, PC2, PC3, PC4, PC5, PC6.

Table S7. Hierarchical linear regression for variance explained in psychotic symptoms by Psychosis PRS

Model		Coef	Std Err	Std Coef	t	p
H_0	(Intercept)	16.14	2.02		7.98	0.000
	age	-0.09	0.02	-0.06	-5.01	0.000
	sex	-0.57	0.25		-2.27	0.023
	PC1	-80.18	10.15	-0.10	-7.90	0.000
	PC2	-42.00	10.14	-0.05	-4.14	0.000
	PC3	-47.60	10.14	-0.06	-4.70	0.000
	PC4	19.33	10.13	0.02	1.91	0.057
	PC5	-2.14	10.13	0.00	-0.21	0.833
	PC6	-3.63	10.14	0.00	-0.36	0.720
H_1	(Intercept)	20.53	2.38		8.62	0.000
	prsPsychosis	0.20	0.06	0.04	3.49	0.000
	age	-0.08	0.02	-0.06	-4.97	0.000
	sex	-0.56	0.25		-2.24	0.025
	PC1	-77.46	10.17	-0.09	-7.61	0.000
	PC2	-44.62	10.16	-0.05	-4.39	0.000
	PC3	-46.65	10.14	-0.06	-4.60	0.000
	PC4	22.44	10.16	0.03	2.21	0.027
	PC5	-5.90	10.18	-0.01	-0.58	0.562
	PC6	-4.98	10.13	-0.01	-0.49	0.623

Model	R^2	Adjusted R^2	R^2 Change	df1	df2	p
H_0	0.021	0.02	0.021	8	6526	0.000
H_1	0.023	0.021	0.002	1	6525	0.000

Notes. Null model includes age, sex, PC1, PC2, PC3, PC4, PC5, PC6.

Table S8. Canonical loadings for mental health PRS scores and phenotypes

			Pearson's r	Lower 95% CI	Upper 95% CI	p	
Canonical Variate 1							
CV1prs	-	prsADHD	-0.87	-0.88	-0.87	0.000	***
CV1prs	-	prsAnxiety	-0.35	-0.37	-0.33	0.000	***
CV1prs	-	prsDepression	-0.69	-0.71	-0.68	0.000	***
CV1prs	-	prsPsychosis	-0.35	-0.37	-0.33	0.000	***
CV1prs	-	phenoADHD	-0.11	-0.14	-0.09	0.000	***
CV1prs	-	phenoAnxiety	-0.05	-0.07	-0.02	0.000	***
CV1prs	-	phenoDepression	-0.07	-0.09	-0.05	0.000	***
CV1prs	-	phenoPsychosis	-0.12	-0.14	-0.09	0.000	***
CV1pheno	-	prsADHD	-0.13	-0.16	-0.11	0.000	***
CV1pheno	-	prsAnxiety	-0.05	-0.08	-0.03	0.000	***
CV1pheno	-	prsDepression	-0.11	-0.13	-0.08	0.000	***
CV1pheno	-	prsPsychosis	-0.05	-0.08	-0.03	0.000	***
CV1pheno	-	phenoADHD	-0.74	-0.75	-0.73	0.000	***
CV1pheno	-	phenoAnxiety	-0.31	-0.33	-0.29	0.000	***
CV1pheno	-	phenoDepression	-0.46	-0.48	-0.44	0.000	***
CV1pheno	-	phenoPsychosis	-0.77	-0.78	-0.76	0.000	***
Canonical Variate 2							
CV2prs	-	prsADHD	0.40	0.38	0.42	0.000	***
CV2prs	-	prsAnxiety	-0.51	-0.52	-0.49	0.000	***
CV2prs	-	prsDepression	-0.67	-0.68	-0.66	0.000	***
CV2prs	-	prsPsychosis	-0.12	-0.15	-0.10	0.000	***
CV2prs	-	phenoADHD	0.04	0.02	0.06	0.001	**
CV2prs	-	phenoAnxiety	-0.04	-0.06	-0.01	0.002	**
CV2prs	-	phenoDepression	-0.04	-0.06	-0.01	0.004	**
CV2prs	-	phenoPsychosis	-0.04	-0.06	-0.01	0.004	**
CV2pheno	-	prsADHD	0.04	0.01	0.06	0.003	**
CV2pheno	-	prsAnxiety	-0.05	-0.07	-0.02	0.000	***
CV2pheno	-	prsDepression	-0.06	-0.09	-0.04	0.000	***
CV2pheno	-	prsPsychosis	-0.01	-0.04	0.01	0.364	
CV2pheno	-	phenoADHD	0.42	0.40	0.44	0.000	***
CV2pheno	-	phenoAnxiety	-0.41	-0.43	-0.38	0.000	***
CV2pheno	-	phenoDepression	-0.38	-0.40	-0.36	0.000	***
CV2pheno	-	phenoPsychosis	-0.38	-0.40	-0.36	0.000	***

Notes. CV1= First canonical variate. CV2=Second canonical variate. CV#prs= Canonical component for PRS scores. CV#pheno= Canonical component for phenotypes. * p < .05, ** p < .01, *** p < .001.

Table S9. Hierarchical linear regression for variance explained in ADHD symptoms by the PRS component scores

Model		Coef	Std Err	Std Coef	t	p
H_0	*(Intercept)*	5.05	0.59		8.58	0.000
	age	-0.02	0.01	-0.04	-3.29	0.001
	sex	-0.99	0.07		-13.54	0.000
	PC1	-12.63	2.95	-0.05	-4.28	0.000
	PC2	9.91	2.95	0.04	3.36	0.001
	PC3	-3.63	2.95	-0.02	-1.23	0.218
	PC4	-8.71	2.95	-0.04	-2.96	0.003
	PC5	-5.21	2.95	-0.02	-1.77	0.077
	PC6	-0.14	2.95	0.00	-0.05	0.962
H_1	*(Intercept)*	5.08	0.59		8.69	0.000
	CV1prs	0.35	0.04	0.12	8.66	0.000
	CV2prs	0.09	0.04	0.03	2.44	0.015
	age	-0.02	0.01	-0.04	-3.35	0.001
	sex	-1.00	0.07		-13.73	0.000
	PC1	-2.51	3.30	-0.01	-0.76	0.447
	PC2	10.44	3.02	0.04	3.45	0.001
	PC3	-5.83	2.94	-0.02	-1.99	0.047
	PC4	-7.28	2.96	-0.03	-2.46	0.014
	PC5	-7.93	2.94	-0.03	-2.70	0.007
	PC6	-0.95	2.93	0.00	-0.33	0.745
**Model**	**R^2**	**Adjusted R^2**	**R^2 Change**	**df1**	**df2**	**p**
H_0	0.035	0.034	0.035	8	6524	0.000
H_1	0.048	0.046	0.013	2	6522	0.000

Notes. CV1prs= First canonical component for PRS scores. CV2prs= Second canonical component for PRS scores. Null model includes age, sex, PC1, PC2, PC3, PC4, PC5, PC6.

Table S10. Hierarchical linear regression for variance explained in Anxiety symptoms by the PRS component scores

Model		Coef	Std Err	Std Coef	t	p
H_0	*(Intercept)*	2.76	0.50		5.58	0.000
	age	-0.01	0.00	-0.02	-1.19	0.233
	sex	-0.03	0.06		-0.42	0.675
	PC1	-1.74	2.48	-0.01	-0.70	0.485
	PC2	1.60	2.48	0.01	0.65	0.518
	PC3	-5.56	2.48	-0.03	-2.25	0.025
	PC4	-0.17	2.48	0.00	-0.07	0.946
	PC5	-4.29	2.48	-0.02	-1.73	0.083
	PC6	-4.03	2.48	-0.02	-1.63	0.104
H_1	*(Intercept)*	2.81	0.49		5.70	0.000
	CV1prs	0.16	0.03	0.07	4.70	0.000
	CV2prs	-0.12	0.03	-0.05	-3.54	0.000
	age	-0.01	0.00	-0.02	-1.30	0.193
	sex	-0.03	0.06		-0.42	0.673
	PC1	5.32	2.79	0.03	1.91	0.056
	PC2	4.58	2.55	0.02	1.79	0.073
	PC3	-6.27	2.48	-0.03	-2.53	0.011
	PC4	-1.42	2.50	-0.01	-0.57	0.570
	PC5	-4.83	2.48	-0.02	-1.95	0.052
	PC6	-4.28	2.47	-0.02	-1.73	0.084

Model	R^2	Adjusted R^2	R^2 Change	df1	df2	p
H_0	0.045	0.002	0.002	8	6524	0.100
H_1	0.083	0.007	0.005	2	6522	0.000

Notes. CV1prs= First canonical component for PRS scores. CV2prs= Second canonical component for PRS scores. Null model includes age, sex, PC1, PC2, PC3, PC4, PC5, PC6.

Table S11. Hierarchical linear regression for variance explained in Depression symptoms by the PRS component scores

Model		Coef	Std Err	Std Coef	t	p
H_0	*(Intercept)*	0.91	0.41		2.25	0.024
	age	0.00	0.00	0.02	1.32	0.186
	sex	-0.27	0.05		-5.36	0.000
	PC1	-4.67	2.03	-0.03	-2.30	0.021
	PC2	3.09	2.03	0.02	1.53	0.127
	PC3	-3.99	2.03	-0.02	-1.97	0.049
	PC4	-0.21	2.03	0.00	-0.10	0.917
	PC5	-3.95	2.03	-0.02	-1.95	0.051
	PC6	-1.82	2.03	-0.01	-0.90	0.371
H_1	*(Intercept)*	0.96	0.40		2.38	0.018
	CV1prs	0.17	0.03	0.08	6.06	0.000
	CV2prs	-0.09	0.03	-0.04	-3.33	0.001
	age	0.00	0.00	0.02	1.21	0.227
	sex	-0.27	0.05		-5.39	0.000
	PC1	2.27	2.28	0.01	1.00	0.320
	PC2	5.66	2.08	0.03	2.72	0.007
	PC3	-4.80	2.03	-0.03	-2.37	0.018
	PC4	-1.14	2.04	-0.01	-0.56	0.579
	PC5	-4.67	2.03	-0.03	-2.30	0.022
	PC6	-2.10	2.02	-0.01	-1.04	0.298

Model	R^2	Adjusted R^2	R^2 Change	df1	df2	p
H_0	0.007	0.006	0.007	8	6524	0.000
H_1	0.014	0.012	0.007	2	6522	0.000

Notes. CV1prs= First canonical component for PRS scores. CV2prs= Second canonical component for PRS scores. Null model includes age, sex, PC1, PC2, PC3, PC4, PC5, PC6.

Table S12. Hierarchical linear regression for variance explained in Psychosis symptoms by the PRS component scores

Model		Coef	Std Err	Std Coef	t	p
H_0	*(Intercept)*	16.13	2.02		7.97	0.000
	age	-0.09	0.02	-0.06	-5.00	0.000
	sex	-0.57	0.25		-2.26	0.024
	PC1	-80.09	10.16	-0.10	-7.89	0.000
	PC2	-41.84	10.15	-0.05	-4.12	0.000
	PC3	-47.52	10.14	-0.06	-4.69	0.000
	PC4	19.34	10.14	0.02	1.91	0.056
	PC5	-2.17	10.13	0.00	-0.21	0.830
	PC6	-3.60	10.14	0.00	-0.36	0.723
H_1	*(Intercept)*	16.31	2.02		8.09	0.000
	CV1prs	1.00	0.14	0.10	7.17	0.000
	CV2prs	-0.11	0.13	-0.01	-0.85	0.397
	age	-0.09	0.02	-0.06	-5.11	0.000
	sex	-0.58	0.25		-2.32	0.021
	PC1	-45.36	11.38	-0.06	-3.98	0.000
	PC2	-33.77	10.42	-0.04	-3.24	0.001
	PC3	-53.09	10.14	-0.06	-5.24	0.000
	PC4	18.83	10.22	0.02	1.84	0.066
	PC5	-8.24	10.15	-0.01	-0.81	0.417
	PC6	-5.63	10.11	-0.01	-0.56	0.578

Model	R^2	Adjusted R^2	R^2 Change	df1	df2	p
H_0	0.021	0.020	0.021	8	6524	0.000
H_1	0.028	0.027	0.008	2	6522	0.000

Notes. CV1prs= First canonical component for PRS scores. CV2prs= Second canonical component for PRS scores. Null model includes age, sex, PC1, PC2, PC3, PC4, PC5, PC6.

Table S13. Association between the first PRS component scores and cumulative adversity

	Coef	Std Err	Std Coef	t	Lower 95% CI	Upper 95% CI	p
(Intercept)	1.670	0.357		4.681	0.971	2.370	0.000
CV1prs	0.166	0.025	0.089	6.762	0.118	0.215	0.000
CV1pheno	0.447	0.023	0.239	19.800	0.403	0.492	0.000
age	-0.002	0.003	-0.010	-0.812	-0.008	0.003	0.417
sex	0.129	0.045		2.888	0.041	0.216	0.004
PC1	-18.741	1.959	-0.124	-9.566	-22.581	-14.901	0.000
PC2	10.671	1.791	0.070	5.958	7.160	14.182	0.000
PC3	-10.877	1.792	-0.072	-6.071	-14.389	-7.365	0.000
PC4	-0.521	1.773	-0.003	-0.294	-3.996	2.954	0.769
PC5	-14.017	1.789	-0.093	-7.836	-17.524	-10.511	0.000
PC6	-4.093	1.781	-0.027	-2.298	-7.584	-0.601	0.022

Notes. CV1prs= First canonical component for PRS scores. CV1pheno= First canonical component for phenotype scores.

Table S14. Association between the second PRS component scores and cumulative adversity

	Coef	Std Err	Std Coef	t	Lower 95% CI	Upper 95% CI	p
(Intercept)	2.213	0.368		6.019	1.492	2.934	0.000
CV2prs	0.000	0.024	0.000	-0.012	-0.048	0.047	0.991
CV2pheno	-0.175	0.023	-0.093	-7.574	-0.220	-0.130	0.000
age	-0.006	0.003	-0.025	-2.071	-0.012	0.000	0.038
sex	-0.025	0.046		-0.545	-0.115	0.065	0.586
PC1	-27.744	1.877	-0.183	-14.777	-31.424	-24.063	0.000
PC2	10.035	1.891	0.066	5.307	6.328	13.741	0.000
PC3	-11.177	1.842	-0.074	-6.067	-14.789	-7.566	0.000
PC4	-1.625	1.854	-0.011	-0.876	-5.260	2.011	0.381
PC5	-13.520	1.842	-0.089	-7.339	-17.132	-9.909	0.000
PC6	-3.654	1.839	-0.024	-1.987	-7.258	-0.049	0.047

Notes. CV2prs= Second canonical component for PRS scores. CV1pheno= Second canonical component for phenotype scores.

Table S15. Association between PRS ADHD and cumulative adversity

	Coef	Std Err	Std Coef	t	Lower 95% CI	Upper 95% CI	p
(Intercept)	3.376	0.486		6.947	2.423	4.328	0.000
prsADHD	0.042	0.007	0.077	6.123	0.028	0.055	0.000
phenoADHD	0.148	0.008	0.238	19.724	0.134	0.163	0.000
age	-0.004	0.003	-0.014	-1.219	-0.009	0.002	0.223
sex	0.158	0.045		3.519	0.070	0.246	0.000
PC1	-22.549	1.868	-0.149	-12.074	-26.210	-18.888	0.000
PC2	7.828	1.789	0.052	4.377	4.322	11.334	0.000
PC3	-12.082	1.793	-0.080	-6.737	-15.598	-8.567	0.000
PC4	0.935	1.779	0.006	0.525	-2.553	4.422	0.599
PC5	-13.612	1.791	-0.090	-7.601	-17.123	-10.102	0.000
PC6	-4.081	1.784	-0.027	-2.288	-7.577	-0.585	0.022

Table S16. Association between PRS Anxiety and cumulative adversity

	Coef	Std Err	Std Coef	t	Lower 95% CI	Upper 95% CI	p
(Intercept)	3.179	0.457		6.956	2.283	4.074	0.000
prsAnxiety	0.039	0.007	0.065	5.272	0.025	0.054	0.000
phenoAnxiety	0.180	0.009	0.237	20.061	0.162	0.197	0.000
age	-0.006	0.003	-0.022	-1.871	-0.011	0.000	0.061
sex	0.016	0.044		0.371	-0.070	0.103	0.710
PC1	-25.045	1.844	-0.165	-13.584	-28.659	-21.431	0.000
PC2	10.264	1.806	0.068	5.682	6.723	13.804	0.000
PC3	-11.066	1.790	-0.073	-6.181	-14.575	-7.556	0.000
PC4	-1.825	1.787	-0.012	-1.021	-5.327	1.678	0.307
PC5	-12.511	1.787	-0.083	-7.000	-16.015	-9.007	0.000
PC6	-2.962	1.786	-0.020	-1.658	-6.464	0.540	0.097

Table S17. Association between PRS Depression and cumulative adversity

	Coef	Std Err	Std Coef	t	Lower 95% CI	Upper 95% CI	p
(Intercept)	3.396	0.486		6.980	2.442	4.349	0.000
prsDepression	0.046	0.010	0.059	4.413	0.025	0.066	0.000
phenoDepression	0.267	0.011	0.289	24.737	0.246	0.288	0.000
age	-0.008	0.003	-0.030	-2.559	-0.013	-0.002	0.011
sex	0.089	0.044		2.037	0.003	0.175	0.042
PC1	-22.779	1.950	-0.151	-11.683	-26.601	-18.957	0.000
PC2	10.398	1.814	0.069	5.733	6.843	13.953	0.000
PC3	-10.865	1.763	-0.072	-6.163	-14.321	-7.409	0.000
PC4	-1.672	1.761	-0.011	-0.949	-5.124	1.781	0.343
PC5	-12.518	1.760	-0.083	-7.111	-15.969	-9.067	0.000
PC6	-3.273	1.759	-0.022	-1.861	-6.722	0.176	0.063

Table S18. Association between PRS Psychosis and cumulative adversity

	Coef	Std Err	Std Coef	t	Lower 95% CI	Upper 95% CI	p
(Intercept)	2.352	0.433		5.429	1.503	3.201	0.000
prsPsychosis	0.026	0.010	0.031	2.528	0.006	0.046	0.011
phenoPsychosis	0.023	0.002	0.123	10.082	0.018	0.027	0.000
age	-0.004	0.003	-0.016	-1.280	-0.010	0.002	0.201
sex	0.029	0.045		0.631	-0.060	0.118	0.528
PC1	-25.726	1.847	-0.170	-13.932	-29.346	-22.106	0.000
PC2	9.944	1.842	0.066	5.398	6.333	13.555	0.000
PC3	-10.496	1.837	-0.069	-5.715	-14.097	-6.896	0.000
PC4	-1.006	1.830	-0.007	-0.550	-4.594	2.581	0.582
PC5	-14.029	1.842	-0.093	-7.615	-17.640	-10.417	0.000
PC6	-4.023	1.832	-0.027	-2.196	-7.614	-0.432	0.028

Table S19. Association of the first PRS component score, adversity, and their interaction with the first phenotype component score

Model		Coef	Std Err	Std Coef	t	Lower 95% CI	Upper 95% CI	p
H_0	*(Intercept)*	0.844	0.192		4.393	0.468	1.221	0.000
	CV1prs	0.116	0.013	0.116	8.768	0.090	0.142	0.000
	adversity	0.130	0.007	0.243	19.800	0.117	0.143	0.000
	age	-0.008	0.002	-0.056	-4.754	-0.011	-0.004	0.000
	sex	-0.262	0.024		-11.005	-0.308	-0.215	0.000
	PC1	-0.697	1.063	-0.009	-0.656	-2.780	1.386	0.512
	PC2	-1.044	0.967	-0.013	-1.080	-2.940	0.852	0.280
	PC3	-3.128	0.967	-0.039	-3.235	-5.023	-1.233	0.001
	PC4	-0.444	0.955	-0.006	-0.465	-2.315	1.428	0.642
	PC5	-0.439	0.968	-0.005	-0.453	-2.336	1.459	0.650
	PC6	-0.280	0.960	-0.003	-0.292	-2.161	1.601	0.770
H_1	*(Intercept)*	0.846	0.192		4.400	0.469	1.223	0.000
	CV1prs	0.123	0.016	0.123	7.744	0.092	0.155	0.000
	adversity	0.131	0.007	0.245	19.591	0.118	0.144	0.000
	age	-0.008	0.002	-0.057	-4.760	-0.011	-0.004	0.000
	sex	-0.262	0.024		-10.994	-0.308	-0.215	0.000
	PC1	-0.765	1.066	-0.009	-0.718	-2.854	1.324	0.473
	PC2	-0.975	0.971	-0.012	-1.004	-2.878	0.928	0.315
	PC3	-3.148	0.967	-0.039	-3.255	-5.044	-1.252	0.001
	PC4	-0.438	0.955	-0.005	-0.459	-2.310	1.433	0.646
	PC5	-0.464	0.968	-0.006	-0.479	-2.362	1.435	0.632
	PC6	-0.277	0.960	-0.003	-0.288	-2.158	1.604	0.773
	CV1prs ✻ adversity	-0.005	0.006	-0.013	-0.842	-0.018	0.007	0.400

Model	R	R^2	Adjusted R^2	RMSE	R^2 Change	df1	df2	p
H_0	0.326	0.106	0.105	0.948	0.106	10	6364	0.000
H_1	0.326	0.106	0.105	0.948	0.000	1	6363	0.400

Notes. CV1prs= First canonical component for PRS scores. H_1 model includes interaction between CV1prs and adversity.

Table S20. Association of the second PRS component score, adversity, and their interaction with the second phenotype component score

Model		Coef	Std Err	Std Coef	t	Lower 95% CI	Upper 95% CI	p
H_0	*(Intercept)*	0.515	0.199		2.589	0.125	0.905	0.010
	CV2prs	0.084	0.013	0.084	6.409	0.058	0.109	0.000
	adversity	-0.051	0.007	-0.096	-7.574	-0.064	-0.038	0.000
	age	-0.003	0.002	-0.021	-1.667	-0.006	0.000	0.096
	sex	-0.230	0.025		-9.349	-0.279	-0.182	0.000
	PC1	-1.694	1.031	-0.021	-1.644	-3.715	0.327	0.100
	PC2	2.763	1.023	0.034	2.702	0.758	4.768	0.007
	PC3	2.139	0.997	0.027	2.145	0.184	4.094	0.032
	PC4	-2.541	1.001	-0.032	-2.539	-4.504	-0.579	0.011
	PC5	-0.665	0.999	-0.008	-0.665	-2.623	1.294	0.506
	PC6	1.598	0.993	0.020	1.610	-0.348	3.545	0.108
H_1	*(Intercept)*	0.519	0.199		2.608	0.129	0.909	0.009
	CV2prs	0.057	0.016	0.057	3.529	0.025	0.089	0.000
	adversity	-0.050	0.007	-0.094	-7.378	-0.063	-0.037	0.000
	age	-0.003	0.002	-0.021	-1.691	-0.006	0.000	0.091
	sex	-0.229	0.025		-9.308	-0.277	-0.181	0.000
	PC1	-1.741	1.030	-0.022	-1.690	-3.761	0.279	0.091
	PC2	2.705	1.022	0.033	2.645	0.700	4.709	0.008
	PC3	2.030	0.998	0.025	2.035	0.075	3.986	0.042
	PC4	-2.624	1.001	-0.033	-2.622	-4.586	-0.662	0.009
	PC5	-0.585	0.999	-0.007	-0.586	-2.544	1.373	0.558
	PC6	1.600	0.992	0.020	1.612	-0.345	3.546	0.107
	CV2prs ✻ adversity	0.019	0.007	0.043	2.759	0.005	0.032	0.006

Model	R	R^2	Adjusted R^2	RMSE	R^2 Change	df1	df2	p
H_0	0.185	0.034	0.033	0.981	0.034	10	6364	0.000
H_1	0.189	0.036	0.034	0.981	0.001	1	6363	0.006

Notes. CV2prs= Second canonical component for PRS scores. H_1 model includes interaction between CV2prs and adversity.

Table S21. Association of PRS ADHD, adversity, and their interaction with the ADHD phenotype

Model		Coef	Std Err	Std Coef	t	Lower 95% CI	Upper 95% CI	p
H_0	*(Intercept)*	8.077	0.782		10.326	6.544	9.611	0.000
	prsADHD	0.082	0.011	0.094	7.445	0.060	0.103	0.000
	adversity	0.388	0.020	0.242	19.724	0.349	0.427	0.000
	age	-0.014	0.005	-0.034	-2.845	-0.023	-0.004	0.004
	sex	-1.007	0.072		-14.078	-1.147	-0.867	0.000
	PC1	4.904	3.054	0.020	1.606	-1.082	10.891	0.108
	PC2	5.853	2.895	0.024	2.021	0.177	11.529	0.043
	PC3	-1.431	2.910	-0.006	-0.492	-7.136	4.273	0.623
	PC4	-7.519	2.875	-0.031	-2.615	-13.155	-1.883	0.009
	PC5	-2.253	2.909	-0.009	-0.775	-7.955	3.449	0.439
	PC6	0.547	2.885	0.002	0.190	-5.109	6.203	0.850
H_1	*(Intercept)*	7.861	0.871		9.027	6.154	9.568	0.000
	prsADHD	0.077	0.014	0.089	5.678	0.051	0.104	0.000
	adversity	0.542	0.273	0.338	1.986	0.007	1.077	0.047
	age	-0.014	0.005	-0.034	-2.842	-0.023	-0.004	0.004
	sex	-1.007	0.072		-14.079	-1.148	-0.867	0.000
	PC1	5.007	3.059	0.021	1.637	-0.990	11.004	0.102
	PC2	5.724	2.904	0.024	1.971	0.030	11.418	0.049
	PC3	-1.424	2.910	-0.006	-0.489	-7.129	4.282	0.625
	PC4	-7.545	2.876	-0.031	-2.624	-13.182	-1.908	0.009
	PC5	-2.196	2.911	-0.009	-0.754	-7.901	3.510	0.451
	PC6	0.544	2.885	0.002	0.189	-5.112	6.201	0.850
	prsADHD ✻ adversity	0.003	0.006	0.096	0.566	-0.008	0.015	0.571

Model	R	R^2	Adjusted R^2	RMSE	R^2 Change	df1	df2	p
H_0	0.320	0.103	0.101	2.850	0.103	10	6366	0.000
H_1	0.320	0.103	0.101	2.850	0.000	1	6365	0.571

Notes. H_1 model includes interaction between prsADHD and adversity.

Table S22. Association of PRS Anxiety, adversity, and their interaction with the Anxiety phenotype

Model		Coef	Std Err	Std Coef	t	Lower 95% CI	Upper 95% CI	p
H_0	*(Intercept)*	2.674	0.621		4.307	1.457	3.891	0.000
	prsAnxiety	0.023	0.010	0.029	2.246	0.003	0.042	0.025
	adversity	0.331	0.016	0.251	20.061	0.298	0.363	0.000
	age	-0.001	0.004	-0.003	-0.282	-0.009	0.007	0.778
	sex	-0.025	0.060		-0.423	-0.143	0.092	0.672
	PC1	8.526	2.533	0.043	3.366	3.561	13.490	0.001
	PC2	-0.571	2.454	-0.003	-0.233	-5.383	4.240	0.816
	PC3	-1.884	2.434	-0.009	-0.774	-6.655	2.887	0.439
	PC4	-0.871	2.422	-0.004	-0.360	-5.619	3.877	0.719
	PC5	-0.550	2.432	-0.003	-0.226	-5.318	4.217	0.821
	PC6	-3.500	2.421	-0.018	-1.445	-8.247	1.247	0.148
H_1	*(Intercept)*	3.005	0.681		4.416	1.671	4.340	0.000
	prsAnxiety	0.031	0.013	0.040	2.513	0.007	0.056	0.012
	adversity	0.104	0.191	0.079	0.543	-0.271	0.479	0.587
	age	-0.001	0.004	-0.003	-0.286	-0.009	0.007	0.775
	sex	-0.025	0.060		-0.421	-0.143	0.092	0.673
	PC1	8.474	2.533	0.042	3.345	3.508	13.439	0.001
	PC2	-0.537	2.455	-0.003	-0.219	-5.349	4.274	0.827
	PC3	-1.933	2.434	-0.010	-0.794	-6.704	2.838	0.427
	PC4	-0.909	2.422	-0.005	-0.375	-5.657	3.839	0.707
	PC5	-0.516	2.432	-0.003	-0.212	-5.283	4.252	0.832
	PC6	-3.536	2.422	-0.018	-1.460	-8.283	1.211	0.144
	prsAnxiety ✻ adversity	-0.006	0.005	-0.172	-1.187	-0.016	0.004	0.235

Model	R	R^2	Adjusted R^2	RMSE	R^2 Change	df1	df2	p
H_0	0.252	0.064	0.062	2.393	0.064	10	6366	0.000
H_1	0.253	0.064	0.062	2.392	0.000	1	6365	0.235

Notes. H_1 model includes interaction between prsAnxiety and adversity.

Table S23. Association of PRS Depression, adversity, and their interaction with the Depression phenotype

Model		Coef	Std Err	Std Coef	t	Lower 95% CI	Upper 95% CI	p
H_0	*(Intercept)*	1.806	0.541		3.339	0.746	2.866	0.001
	prsDepression	0.052	0.011	0.061	4.508	0.029	0.074	0.000
	adversity	0.328	0.013	0.303	24.737	0.302	0.354	0.000
	age	0.007	0.003	0.025	2.081	0.000	0.013	0.037
	sex	-0.276	0.048		-5.707	-0.371	-0.181	0.000
	PC1	8.358	2.182	0.051	3.831	4.081	12.636	0.000
	PC2	1.963	2.015	0.012	0.974	-1.988	5.914	0.330
	PC3	-0.680	1.960	-0.004	-0.347	-4.523	3.162	0.728
	PC4	-1.224	1.952	-0.007	-0.627	-5.051	2.603	0.531
	PC5	-0.128	1.959	-0.001	-0.065	-3.968	3.713	0.948
	PC6	-1.843	1.950	-0.011	-0.945	-5.666	1.981	0.345
H_1	*(Intercept)*	1.531	0.593		2.582	0.369	2.694	0.010
	prsDepression	0.043	0.014	0.051	3.153	0.016	0.070	0.002
	adversity	0.520	0.170	0.480	3.051	0.186	0.854	0.002
	age	0.007	0.003	0.025	2.096	0.000	0.013	0.036
	sex	-0.277	0.048		-5.732	-0.372	-0.182	0.000
	PC1	8.516	2.186	0.052	3.895	4.230	12.801	0.000
	PC2	1.863	2.017	0.011	0.924	-2.092	5.818	0.356
	PC3	-0.574	1.962	-0.003	-0.292	-4.420	3.273	0.770
	PC4	-1.188	1.952	-0.007	-0.609	-5.016	2.639	0.543
	PC5	-0.116	1.959	-0.001	-0.059	-3.957	3.724	0.953
	PC6	-1.864	1.951	-0.011	-0.956	-5.688	1.959	0.339
	prsDepression ✻ adversity	0.006	0.005	0.177	1.129	-0.004	0.017	0.259

Model	R	R^2	Adjusted R^2	RMSE	R^2 Change	df1	df2	p
H_0	0.316	0.100	0.099	1.927	0.100	10	6366	0.000
H_1	0.317	0.100	0.099	1.927	0.000	1	6365	0.259

Notes. H_1 model includes interaction between prsDepression and adversity.

Table S24. Association of PRS Psychosis, adversity, and their interaction with the Psychosis phenotype

Model		Coef	Std Err	Std Coef	t	Lower 95% CI	Upper 95% CI	p
H_0	*(Intercept)*	18.267	2.397		7.621	13.568	22.965	0.000
	prsPsychosis	0.166	0.056	0.037	2.959	0.056	0.277	0.003
	adversity	0.695	0.069	0.128	10.082	0.560	0.830	0.000
	age	-0.079	0.017	-0.058	-4.670	-0.112	-0.046	0.000
	sex	-0.533	0.252		-2.118	-1.027	-0.040	0.034
	PC1	-58.792	10.367	-0.071	-5.671	-79.114	-38.470	0.000
	PC2	-48.825	10.219	-0.059	-4.778	-68.857	-28.793	0.000
	PC3	-38.411	10.197	-0.046	-3.767	-58.400	-18.422	0.000
	PC4	21.282	10.143	0.026	2.098	1.398	41.167	0.036
	PC5	3.117	10.260	0.004	0.304	-16.996	23.230	0.761
	PC6	-5.519	10.160	-0.007	-0.543	-25.436	14.397	0.587
H_1	*(Intercept)*	20.919	2.584		8.097	15.854	25.983	0.000
	prsPsychosis	0.285	0.071	0.063	4.017	0.146	0.424	0.000
	adversity	-1.144	0.675	-0.210	-1.696	-2.467	0.178	0.090
	age	-0.079	0.017	-0.057	-4.647	-0.112	-0.046	0.000
	sex	-0.533	0.252		-2.116	-1.026	-0.039	0.034
	PC1	-59.836	10.368	-0.072	-5.771	-80.162	-39.511	0.000
	PC2	-47.153	10.232	-0.057	-4.609	-67.211	-27.095	0.000
	PC3	-38.122	10.192	-0.046	-3.740	-58.102	-18.142	0.000
	PC4	21.731	10.140	0.026	2.143	1.854	41.608	0.032
	PC5	2.548	10.257	0.003	0.248	-17.559	22.654	0.804
	PC6	-5.955	10.156	-0.007	-0.586	-25.864	13.954	0.558
	prsPsychosis ✻ adversity	-0.081	0.030	-0.339	-2.741	-0.139	-0.023	0.006

Model	R	R^2	Adjusted R^2	RMSE	R^2 Change	df1	df2	p
H_0	0.194	0.037	0.036	10.031	0.037	10	6365	0.000
H_1	0.196	0.039	0.037	10.026	0.001	1	6364	0.006

Notes. H_1 model includes interaction between prsPsychosis and adversity.

Figure S6. Prediction of mental health phenotypes by PRS components, adversity, and their interaction

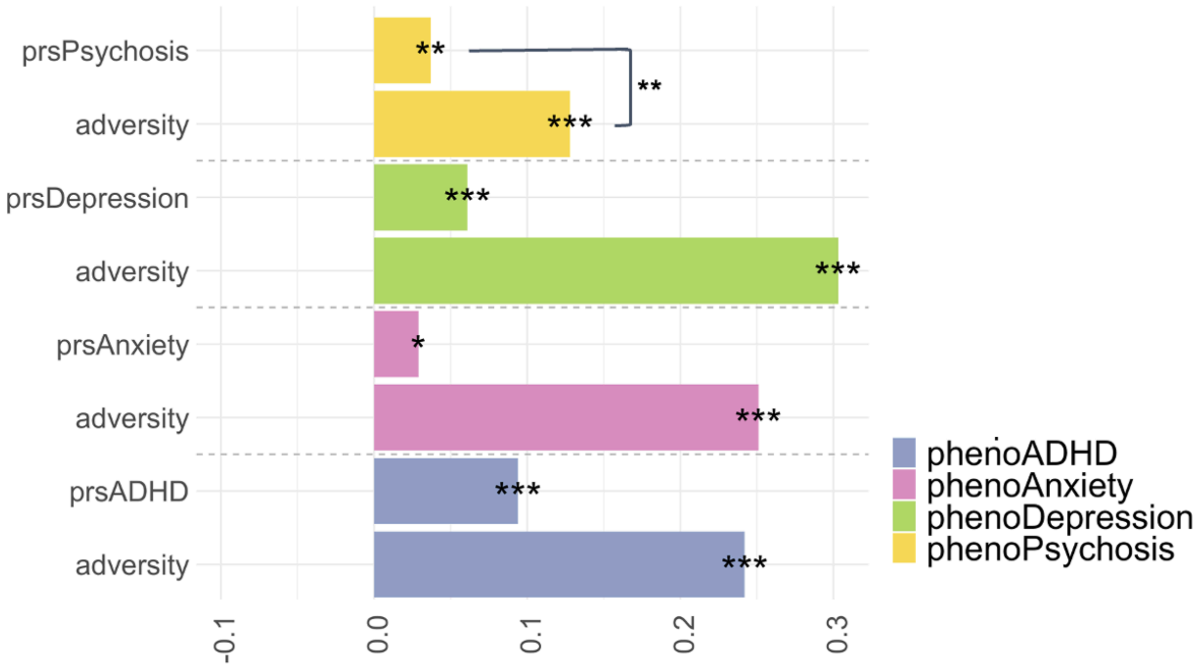

Notes. Regressions model the predictive value for each phenotype separately, including age, sex, and the first 6 population components (PCs) as covariates. Y-axis represents standardised coefficients. Only significant interactions shown.

Table S25. Association between functional connectome clustering and the first PRS component, and its interaction with adversity

Model		Coef	Std Err	Std Coef	t	Lower 95% CI	Upper 95% CI	p
H_0	*(Intercept)*	0.221	0.008		28.451	0.206	0.236	0.000
	CV1prs	-0.001	0.001	-0.021	-1.129	-0.002	0.000	0.259
	CV1pheno	-0.002	0.001	-0.070	-3.988	-0.003	-0.001	0.000
	adversity	0.000	0.000	0.003	0.183	-0.001	0.001	0.855
	age	0.000	0.000	0.087	5.184	0.000	0.000	0.000
	sex	0.008	0.001		8.667	0.006	0.010	0.000
	PC1	0.259	0.045	0.107	5.772	0.171	0.347	0.000
	PC2	0.014	0.042	0.005	0.323	-0.069	0.097	0.747
	PC3	0.024	0.041	0.010	0.576	-0.057	0.104	0.565
	PC4	-0.016	0.039	-0.007	-0.401	-0.092	0.061	0.689
	PC5	-0.034	0.039	-0.014	-0.854	-0.111	0.044	0.393
	PC6	-0.089	0.038	-0.039	-2.320	-0.164	-0.014	0.020
H_1	*(Intercept)*	0.221	0.008		28.442	0.206	0.236	0.000
	CV1prs	-0.001	0.001	-0.024	-1.078	-0.002	0.001	0.281
	CV1pheno	-0.002	0.001	-0.070	-3.980	-0.003	-0.001	0.000
	adversity	0.000	0.000	0.003	0.155	-0.001	0.001	0.877
	age	0.000	0.000	0.087	5.185	0.000	0.000	0.000
	sex	0.008	0.001		8.667	0.006	0.010	0.000
	PC1	0.260	0.045	0.107	5.766	0.172	0.348	0.000
	PC2	0.013	0.043	0.005	0.300	-0.071	0.096	0.764
	PC3	0.024	0.041	0.010	0.582	-0.057	0.105	0.561
	PC4	-0.016	0.039	-0.007	-0.400	-0.092	0.061	0.689
	PC5	-0.033	0.039	-0.014	-0.844	-0.111	0.044	0.399
	PC6	-0.089	0.038	-0.039	-2.326	-0.165	-0.014	0.020
	CV1prs ✻ adversity	0.000	0.000	0.005	0.241	0.000	0.001	0.809

Model	R	R^2	Adjusted R^2	RMSE	R^2 Change	df1	df2	p
H_0	0.233	0.054	0.051	0.028	0.054	11	3377	0.000
H_1	0.233	0.054	0.051	0.028	0	1	3376	0.809

Notes. CV1prs= First canonical component for PRS scores. CV1pheno= First canonical component for phenotype scores. H_1 model includes interaction between CV1prs and adversity.

Table S26. Association between functional connectome modularity and the first PRS component, and its interaction with adversity

Model		Coef	Std Err	Std Coef	t	Lower 95% CI	Upper 95% CI	p
H_0	*(Intercept)*	0.437	0.011		39.248	0.415	0.458	0.000
	CV1prs	0.000	0.001	-0.007	-0.356	-0.002	0.001	0.722
	CV1pheno	-0.002	0.001	-0.049	-2.777	-0.004	-0.001	0.006
	adversity	0.000	0.000	0.019	1.045	0.000	0.001	0.296
	age	0.000	0.000	0.059	3.523	0.000	0.001	0.000
	sex	0.014	0.001		10.477	0.012	0.017	0.000
	PC1	0.094	0.064	0.027	1.466	-0.032	0.220	0.143
	PC2	0.029	0.061	0.008	0.480	-0.090	0.148	0.631
	PC3	-0.037	0.059	-0.011	-0.620	-0.152	0.079	0.535
	PC4	-0.072	0.056	-0.022	-1.297	-0.182	0.037	0.195
	PC5	-0.128	0.056	-0.039	-2.276	-0.239	-0.018	0.023
	PC6	-0.139	0.055	-0.043	-2.526	-0.247	-0.031	0.012
H_1	*(Intercept)*	0.437	0.011		39.257	0.415	0.459	0.000
	CV1prs	0.000	0.001	0.005	0.244	-0.002	0.002	0.808
	CV1pheno	-0.002	0.001	-0.050	-2.798	-0.004	-0.001	0.005
	adversity	0.000	0.000	0.021	1.150	0.000	0.001	0.250
	age	0.000	0.000	0.059	3.515	0.000	0.001	0.000
	sex	0.014	0.001		10.473	0.012	0.017	0.000
	PC1	0.088	0.065	0.025	1.356	-0.039	0.214	0.175
	PC2	0.035	0.061	0.010	0.568	-0.085	0.154	0.570
	PC3	-0.038	0.059	-0.011	-0.647	-0.154	0.078	0.518
	PC4	-0.073	0.056	-0.022	-1.301	-0.182	0.037	0.193
	PC5	-0.131	0.056	-0.039	-2.313	-0.241	-0.020	0.021
	PC6	-0.138	0.055	-0.042	-2.499	-0.245	-0.030	0.012
	CV1prs ✻ adversity	0.000	0.000	-0.021	-0.992	-0.001	0.000	0.321

Model	R	R^2	Adjusted R^2	RMSE	R^2 Change	df1	df2	p
H_0	0.209	0.044	0.040	0.040	0.044	11	3377	0.000
H_1	0.209	0.044	0.040	0.040	0.000	1	3376	0.321

Notes. CV1prs= First canonical component for PRS scores. CV1pheno= First canonical component for phenotype scores. H_1 model includes interaction between CV1prs and adversity.

Table S27. Association between functional connectome assortativity and the first PRS component, and its interaction with adversity

Model		Coef	Std Err	Std Coef	t	Lower 95% CI	Upper 95% CI	p
H_0	*(Intercept)*	-0.044	0.014		-3.109	-0.072	-0.016	0.002
	CV1prs	0.001	0.001	0.011	0.554	-0.001	0.002	0.580
	CV1pheno	0.001	0.001	0.021	1.166	-0.001	0.003	0.244
	adversity	0.001	0.001	0.047	2.584	0.000	0.002	0.010
	age	0.000	0.000	-0.031	-1.803	0.000	0.000	0.071
	sex	0.002	0.002		1.002	-0.002	0.005	0.317
	PC1	-0.380	0.082	-0.087	-4.612	-0.541	-0.218	0.000
	PC2	-0.105	0.078	-0.023	-1.351	-0.257	0.047	0.177
	PC3	-0.040	0.076	-0.009	-0.530	-0.188	0.108	0.596
	PC4	0.018	0.072	0.004	0.257	-0.122	0.159	0.797
	PC5	0.224	0.072	0.053	3.101	0.082	0.366	0.002
	PC6	0.201	0.070	0.049	2.852	0.063	0.339	0.004
H_1	*(Intercept)*	-0.044	0.014		-3.102	-0.072	-0.016	0.002
	CV1prs	0.001	0.001	0.017	0.755	-0.001	0.003	0.450
	CV1pheno	0.001	0.001	0.021	1.153	-0.001	0.003	0.249
	adversity	0.001	0.001	0.048	2.628	0.000	0.002	0.009
	age	0.000	0.000	-0.031	-1.807	0.000	0.000	0.071
	sex	0.002	0.002		0.999	-0.002	0.005	0.318
	PC1	-0.384	0.083	-0.088	-4.642	-0.546	-0.222	0.000
	PC2	-0.101	0.078	-0.022	-1.298	-0.254	0.052	0.194
	PC3	-0.041	0.076	-0.009	-0.544	-0.189	0.107	0.587
	PC4	0.018	0.072	0.004	0.255	-0.122	0.159	0.799
	PC5	0.223	0.072	0.053	3.077	0.081	0.364	0.002
	PC6	0.202	0.070	0.049	2.865	0.064	0.340	0.004
	CV1prs ✻ adversity	0.000	0.000	-0.012	-0.533	-0.001	0.001	0.594

Model	R	R^2	Adjusted R^2	RMSE	R^2 Change	df1	df2	p
H_0	0.144	0.021	0.017	0.051	0.021	11	3377	0.000
H_1	0.144	0.021	0.017	0.051	0.000	1	3376	0.594

Notes. CV1prs= First canonical component for PRS scores. CV1pheno= First canonical component for phenotype scores. H_1 model includes interaction between CV1prs and adversity.

Table S28. Association between functional connectome clustering and the second PRS component, and its interaction with adversity

Model		Coef	Std Err	Std Coef	t	Lower 95% CI	Upper 95% CI	p
H_0	*(Intercept)*	0.220	0.008		28.255	0.204	0.235	0.000
	CV2prs	-0.001	0.001	-0.024	-1.371	-0.002	0.000	0.170
	CV2pheno	0.000	0.001	-0.001	-0.083	-0.001	0.001	0.934
	adversity	0.000	0.000	-0.017	-0.994	-0.001	0.000	0.320
	age	0.000	0.000	0.091	5.397	0.000	0.000	0.000
	sex	0.009	0.001		9.063	0.007	0.011	0.000
	PC1	0.299	0.042	0.123	7.069	0.216	0.382	0.000
	PC2	0.034	0.043	0.014	0.787	-0.051	0.119	0.431
	PC3	0.026	0.041	0.011	0.636	-0.054	0.107	0.525
	PC4	-0.024	0.040	-0.010	-0.610	-0.102	0.053	0.542
	PC5	-0.034	0.039	-0.014	-0.854	-0.111	0.044	0.393
	PC6	-0.093	0.038	-0.041	-2.411	-0.168	-0.017	0.016
H_1	*(Intercept)*	0.220	0.008		28.233	0.204	0.235	0.000
	CV2prs	0.000	0.001	-0.016	-0.731	-0.002	0.001	0.465
	CV2pheno	0.000	0.001	-0.001	-0.035	-0.001	0.001	0.972
	adversity	0.000	0.000	-0.018	-1.014	-0.001	0.000	0.310
	age	0.000	0.000	0.091	5.409	0.000	0.000	0.000
	sex	0.009	0.001		9.070	0.007	0.011	0.000
	PC1	0.301	0.042	0.124	7.098	0.218	0.384	0.000
	PC2	0.034	0.043	0.014	0.792	-0.051	0.119	0.428
	PC3	0.027	0.041	0.011	0.662	-0.053	0.108	0.508
	PC4	-0.023	0.040	-0.010	-0.587	-0.101	0.054	0.557
	PC5	-0.035	0.039	-0.015	-0.879	-0.112	0.043	0.379
	PC6	-0.093	0.038	-0.040	-2.409	-0.168	-0.017	0.016
	CV2prs ✻ adversity	0.000	0.000	-0.015	-0.699	-0.001	0.000	0.485

Model	R	R^2	Adjusted R^2	RMSE	R^2 Change	df1	df2	p
H_0	0.222	0.049	0.046	0.028	0.028	11	3377	0.000
H_1	0.223	0.050	0.046	0.028	0.028	1	3376	0.485

Notes. CV2prs= Second canonical component for PRS scores. CV2pheno= Second canonical component for phenotype scores. H_1 model includes interaction between CV2prs and adversity.

Table S29. Association between functional connectome modularity and the second PRS component, and its interaction with adversity

Model		Coef	Std Err	Std Coef	t	Lower 95% CI	Upper 95% CI	p
H_0	*(Intercept)*	0.435	0.011		39.113	0.413	0.457	0.000
	CV2prs	0.000	0.001	-0.008	-0.466	-0.002	0.001	0.641
	CV2pheno	0.000	0.001	0.008	0.468	-0.001	0.002	0.640
	adversity	0.000	0.000	0.006	0.356	-0.001	0.001	0.722
	age	0.000	0.000	0.062	3.689	0.000	0.001	0.000
	sex	0.015	0.001		10.808	0.012	0.018	0.000
	PC1	0.118	0.060	0.034	1.955	0.000	0.237	0.051
	PC2	0.040	0.062	0.011	0.645	-0.081	0.161	0.519
	PC3	-0.033	0.059	-0.010	-0.565	-0.148	0.082	0.572
	PC4	-0.075	0.057	-0.023	-1.318	-0.186	0.036	0.188
	PC5	-0.129	0.056	-0.039	-2.278	-0.239	-0.018	0.023
	PC6	-0.143	0.055	-0.044	-2.597	-0.251	-0.035	0.009
H_1	*(Intercept)*	0.435	0.011		39.087	0.413	0.457	0.000
	CV2prs	0.000	0.001	0.001	0.056	-0.002	0.002	0.956
	CV2pheno	0.000	0.001	0.009	0.520	-0.001	0.002	0.603
	adversity	0.000	0.000	0.006	0.333	-0.001	0.001	0.740
	age	0.000	0.000	0.063	3.702	0.000	0.001	0.000
	sex	0.015	0.001		10.815	0.012	0.018	0.000
	PC1	0.121	0.061	0.035	1.999	0.002	0.240	0.046
	PC2	0.040	0.062	0.011	0.651	-0.081	0.162	0.515
	PC3	-0.031	0.059	-0.009	-0.536	-0.147	0.084	0.592
	PC4	-0.073	0.057	-0.022	-1.292	-0.184	0.038	0.197
	PC5	-0.130	0.056	-0.039	-2.305	-0.241	-0.019	0.021
	PC6	-0.143	0.055	-0.044	-2.596	-0.251	-0.035	0.009
	CV2prs ✻ adversity	0.000	0.000	-0.016	-0.772	-0.001	0.000	0.440
**Model**	**R**	**R^2**	**Adjusted R^2**	**RMSE**	**R^2 Change**	**df1**	**df2**	**p**
H_0	0.204	0.041	0.038	0.040	0.041	11	3377	0.000
H_1	0.204	0.042	0.038	0.040	0.000	1	3376	0.440

Notes. CV2prs= Second canonical component for PRS scores. CV2pheno= Second canonical component for phenotype scores. H_1 model includes interaction between CV2prs and adversity.

Table S30. Association between functional connectome assortativity and the second PRS component, and its interaction with adversity

Model		Coef	Std Err	Std Coef	t	Lower 95% CI	Upper 95% CI	p
H_0	*(Intercept)*	-0.043	0.014		-3.028	-0.071	-0.015	0.002
	CV2prs	0.000	0.001	-0.009	-0.489	-0.002	0.001	0.625
	CV2pheno	-0.002	0.001	-0.036	-2.091	-0.004	0.000	0.037
	adversity	0.001	0.001	0.050	2.828	0.000	0.002	0.005
	age	0.000	0.000	-0.032	-1.894	0.000	0.000	0.058
	sex	0.001	0.002		0.649	-0.002	0.005	0.516
	PC1	-0.398	0.077	-0.091	-5.142	-0.549	-0.246	0.000
	PC2	-0.097	0.079	-0.021	-1.223	-0.252	0.058	0.221
	PC3	-0.035	0.075	-0.008	-0.467	-0.182	0.112	0.641
	PC4	0.005	0.072	0.001	0.071	-0.137	0.147	0.944
	PC5	0.230	0.072	0.055	3.189	0.089	0.372	0.001
	PC6	0.209	0.070	0.051	2.975	0.071	0.347	0.003
H_1	*(Intercept)*	-0.043	0.014		-3.012	-0.071	-0.015	0.003
	CV2prs	-0.001	0.001	-0.019	-0.850	-0.003	0.001	0.395
	CV2pheno	-0.002	0.001	-0.037	-2.140	-0.004	0.000	0.032
	adversity	0.001	0.001	0.050	2.850	0.000	0.002	0.004
	age	0.000	0.000	-0.033	-1.908	0.000	0.000	0.056
	sex	0.001	0.002		0.640	-0.002	0.005	0.522
	PC1	-0.402	0.078	-0.092	-5.181	-0.553	-0.250	0.000
	PC2	-0.097	0.079	-0.021	-1.229	-0.252	0.058	0.219
	PC3	-0.037	0.075	-0.008	-0.495	-0.185	0.110	0.620
	PC4	0.003	0.072	0.001	0.045	-0.139	0.145	0.964
	PC5	0.232	0.072	0.055	3.216	0.091	0.374	0.001
	PC6	0.209	0.070	0.051	2.973	0.071	0.347	0.003
	CV2prs ✻ adversity	0.000	0.001	0.017	0.787	-0.001	0.001	0.431

Model	R	R^2	Adjusted R^2	RMSE	R^2 Change	df1	df2	p
H_0	0.147	0.022	0.018	0.051	0.022	11	3377	0.000
H_1	0.147	0.022	0.018	0.051	0.000	1	3376	0.431

Notes. CV2prs= Second canonical component for PRS scores. CV2pheno= Second canonical component for phenotype scores. H_1 model includes interaction between CV2prs and adversity.

Figure S7. Association of functional connectome measures with disorder-specific PRS scores and phenotype scores

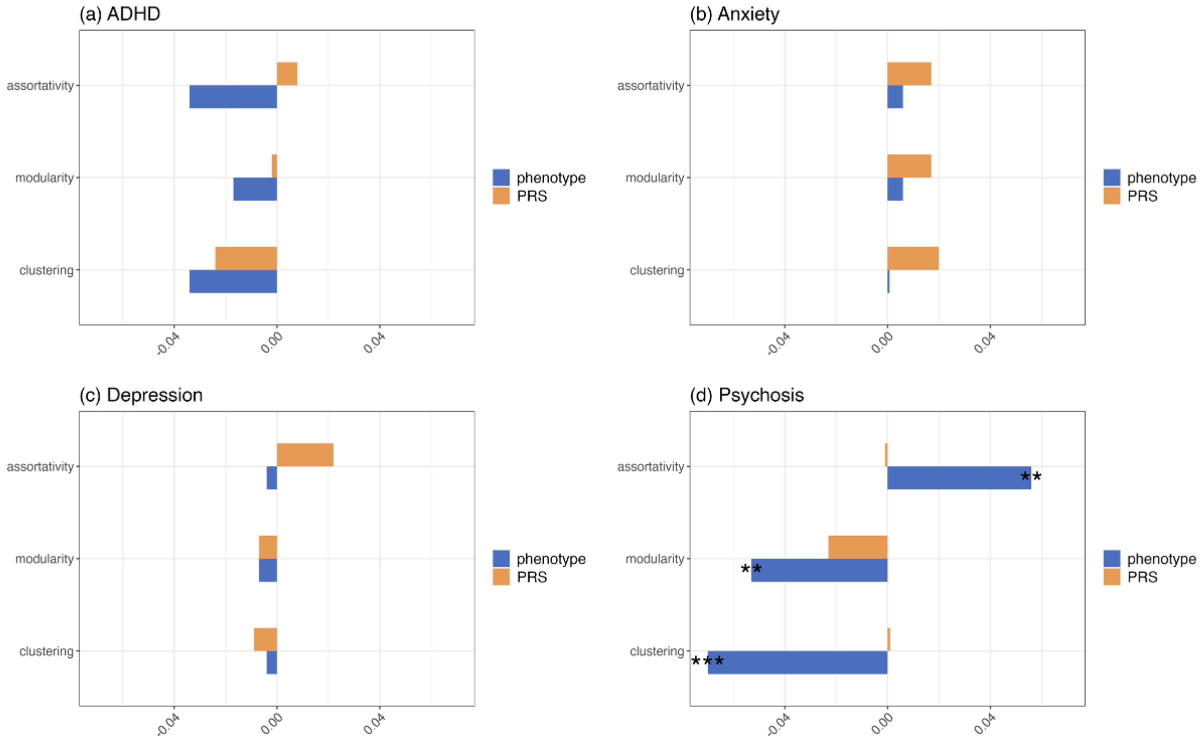

Notes. (A) Variance explained in connectome measures by the ADHD phenotype and ADHD PRS (B) Variance explained in connectome measures by the Anxiety phenotype and Anxiety PRS. (C) Variance explained in connectome measures by the Depression phenotype and Depression PRS. (D) Variance explained in connectome measures by the Psychosis phenotype and Psychosis PRS. Regression models include adversity, age, sex, and the first 6 population components (PCs) as covariates. Y-axis represents standardised coefficients. Bonferroni correction used to adjust the significance cut-off based on the number of brain measures (0.05/3). * $p < .017$, ** $p < .01$, *** $p < .001$.

Table S31. Association between functional connectome clustering and PRS ADHD, and its interaction with adversity

Model		Coef	Std Err	Std Coef	t	Lower 95% CI	Upper 95% CI	p
H_0	*(Intercept)*	0.211	0.011		19.758	0.190	0.232	0.000
	prsADHD	0.000	0.000	-0.024	-1.358	0.000	0.000	0.175
	phenoADHD	0.000	0.000	-0.034	-1.941	-0.001	0.000	0.052
	adversity	0.000	0.000	-0.006	-0.328	-0.001	0.000	0.743
	age	0.000	0.000	0.090	5.368	0.000	0.000	0.000
	sex	0.008	0.001		8.783	0.007	0.010	0.000
	PC1	0.271	0.043	0.112	6.299	0.187	0.355	0.000
	PC2	0.023	0.042	0.009	0.536	-0.060	0.106	0.592
	PC3	0.030	0.041	0.012	0.726	-0.051	0.111	0.468
	PC4	-0.019	0.039	-0.008	-0.492	-0.096	0.057	0.623
	PC5	-0.034	0.039	-0.015	-0.868	-0.112	0.043	0.385
	PC6	-0.091	0.038	-0.040	-2.361	-0.166	-0.015	0.018
H_1	*(Intercept)*	0.212	0.012		18.062	0.189	0.235	0.000
	prsADHD	0.000	0.000	-0.021	-0.977	-0.001	0.000	0.328
	phenoADHD	0.000	0.000	-0.034	-1.936	-0.001	0.000	0.053
	adversity	-0.001	0.004	-0.060	-0.257	-0.008	0.006	0.797
	age	0.000	0.000	0.090	5.368	0.000	0.000	0.000
	sex	0.008	0.001		8.782	0.007	0.010	0.000
	PC1	0.270	0.043	0.112	6.269	0.186	0.355	0.000
	PC2	0.023	0.042	0.009	0.552	-0.060	0.107	0.581
	PC3	0.030	0.041	0.012	0.723	-0.051	0.111	0.469
	PC4	-0.019	0.039	-0.008	-0.490	-0.096	0.058	0.624
	PC5	-0.035	0.040	-0.015	-0.878	-0.112	0.043	0.380
	PC6	-0.091	0.038	-0.040	-2.354	-0.166	-0.015	0.019
	prsADHD ✻ adversity	0.000	0.000	-0.054	-0.233	0.000	0.000	0.816

Model	R	R^2	Adjusted R^2	RMSE	R^2 Change	df1	df2	p
H_0	0.225	0.051	0.048	0.028	0.051	11	3377	0.000
H_1	0.225	0.051	0.047	0.028	0.000	1	3376	0.816

Notes. H_1 model includes interaction between PRS ADHD and adversity.

Table S32. Association between functional connectome modularity and PRS ADHD, and its interaction with adversity

Model		Coef	Std Err	Std Coef	t	Lower 95% CI	Upper 95% CI	p
H_0	*(Intercept)*	0.435	0.015		28.424	0.405	0.465	0.000
	prsADHD	0.000	0.000	-0.002	-0.125	0.000	0.000	0.901
	phenoADHD	0.000	0.000	-0.017	-0.944	-0.001	0.000	0.345
	adversity	0.000	0.000	0.010	0.556	-0.001	0.001	0.579
	age	0.000	0.000	0.062	3.649	0.000	0.001	0.000
	sex	0.015	0.001		10.581	0.012	0.017	0.000
	PC1	0.109	0.062	0.032	1.777	-0.011	0.230	0.076
	PC2	0.036	0.061	0.010	0.592	-0.083	0.155	0.554
	PC3	-0.033	0.059	-0.010	-0.558	-0.149	0.083	0.577
	PC4	-0.074	0.056	-0.022	-1.319	-0.184	0.036	0.187
	PC5	-0.130	0.056	-0.039	-2.307	-0.241	-0.020	0.021
	PC6	-0.142	0.055	-0.044	-2.578	-0.250	-0.034	0.010
H_1	*(Intercept)*	0.446	0.017		26.502	0.413	0.479	0.000
	prsADHD	0.000	0.000	0.017	0.793	0.000	0.001	0.428
	phenoADHD	0.000	0.000	-0.016	-0.912	-0.001	0.000	0.362
	adversity	-0.008	0.005	-0.357	-1.512	-0.019	0.002	0.131
	age	0.000	0.000	0.062	3.653	0.000	0.001	0.000
	sex	0.015	0.001		10.586	0.012	0.017	0.000
	PC1	0.103	0.062	0.030	1.671	-0.018	0.224	0.095
	PC2	0.043	0.061	0.012	0.703	-0.076	0.162	0.482
	PC3	-0.034	0.059	-0.010	-0.575	-0.150	0.082	0.565
	PC4	-0.073	0.056	-0.022	-1.307	-0.183	0.037	0.191
	PC5	-0.134	0.057	-0.040	-2.379	-0.245	-0.024	0.017
	PC6	-0.140	0.055	-0.043	-2.539	-0.248	-0.032	0.011
	prsADHD ✻ adversity	0.000	0.000	-0.365	-1.559	0.000	0.000	0.119

Model	R	R^2	Adjusted R^2	RMSE	R^2 Change	df1	df2	p
H_0	0.204	0.042	0.038	0.040	0.042	11	3377	0.000
H_1	0.206	0.042	0.039	0.040	0.001	1	3376	0.119

Notes. H_1 model includes interaction between PRS ADHD and adversity.

Table S33. Association between functional connectome assortativity and PRS ADHD, and its interaction with adversity

Model		Coef	Std Err	Std Coef	t	Lower 95% CI	Upper 95% CI	p
H_0	*(Intercept)*	-0.036	0.020		-1.842	-0.074	0.002	0.066
	prsADHD	0.000	0.000	0.008	0.433	0.000	0.001	0.665
	phenoADHD	-0.001	0.000	-0.034	-1.871	-0.001	0.000	0.061
	adversity	0.002	0.001	0.061	3.382	0.001	0.003	0.001
	age	0.000	0.000	-0.033	-1.921	0.000	0.000	0.055
	sex	0.001	0.002		0.606	-0.002	0.005	0.544
	PC1	-0.396	0.079	-0.091	-5.022	-0.550	-0.241	0.000
	PC2	-0.108	0.077	-0.024	-1.400	-0.260	0.043	0.162
	PC3	-0.042	0.076	-0.010	-0.561	-0.190	0.106	0.575
	PC4	0.015	0.072	0.004	0.210	-0.125	0.155	0.834
	PC5	0.223	0.072	0.053	3.089	0.082	0.365	0.002
	PC6	0.205	0.070	0.050	2.915	0.067	0.343	0.004
H_1	*(Intercept)*	-0.037	0.022		-1.738	-0.080	0.005	0.082
	prsADHD	0.000	0.000	0.006	0.267	-0.001	0.001	0.789
	phenoADHD	-0.001	0.000	-0.034	-1.874	-0.001	0.000	0.061
	adversity	0.003	0.007	0.097	0.408	-0.011	0.016	0.684
	age	0.000	0.000	-0.033	-1.921	0.000	0.000	0.055
	sex	0.001	0.002		0.606	-0.002	0.005	0.545
	PC1	-0.395	0.079	-0.090	-5.000	-0.550	-0.240	0.000
	PC2	-0.109	0.078	-0.024	-1.407	-0.262	0.043	0.160
	PC3	-0.042	0.076	-0.010	-0.560	-0.190	0.106	0.576
	PC4	0.015	0.072	0.004	0.209	-0.125	0.155	0.835
	PC5	0.224	0.072	0.053	3.092	0.082	0.365	0.002
	PC6	0.205	0.070	0.050	2.910	0.067	0.343	0.004
	prsADHD ✻ adversity	0.000	0.000	0.036	0.151	0.000	0.000	0.880

Model	R	R^2	Adjusted R^2	RMSE	R^2 Change	df1	df2	p
H_0	0.145	0.021	0.018	0.051	0.021	11	3377	0.000
H_1	0.145	0.021	0.018	0.051	0.000	1	3376	0.880

Notes. H_1 model includes interaction between PRS ADHD and adversity.

Table S34. Association between functional connectome clustering and PRS Anxiety, and its interaction with adversity

Model		Coef	Std Err	Std Coef	t	Lower 95% CI	Upper 95% CI	p
H_0	*(Intercept)*	0.227	0.010		22.851	0.208	0.247	0.000
	prsAnxiety	0.000	0.000	0.020	1.163	0.000	0.001	0.245
	phenoAnxiety	0.000	0.000	0.001	0.050	0.000	0.000	0.960
	adversity	0.000	0.000	-0.018	-1.018	-0.001	0.000	0.309
	age	0.000	0.000	0.090	5.367	0.000	0.000	0.000
	sex	0.009	0.001		9.079	0.007	0.011	0.000
	PC1	0.299	0.043	0.123	7.020	0.215	0.382	0.000
	PC2	0.029	0.043	0.011	0.670	-0.055	0.113	0.503
	PC3	0.023	0.041	0.009	0.549	-0.058	0.103	0.583
	PC4	-0.021	0.039	-0.009	-0.528	-0.098	0.056	0.598
	PC5	-0.036	0.039	-0.015	-0.913	-0.113	0.041	0.361
	PC6	-0.094	0.038	-0.041	-2.440	-0.169	-0.018	0.015
H_1	*(Intercept)*	0.228	0.011		21.014	0.207	0.249	0.000
	prsAnxiety	0.000	0.000	0.023	1.056	0.000	0.001	0.291
	phenoAnxiety	0.000	0.000	0.001	0.050	0.000	0.000	0.960
	adversity	-0.001	0.003	-0.056	-0.271	-0.007	0.006	0.786
	age	0.000	0.000	0.090	5.365	0.000	0.000	0.000
	sex	0.009	0.001		9.078	0.007	0.011	0.000
	PC1	0.298	0.043	0.123	7.010	0.215	0.382	0.000
	PC2	0.029	0.043	0.011	0.672	-0.055	0.113	0.502
	PC3	0.022	0.041	0.009	0.545	-0.058	0.103	0.586
	PC4	-0.021	0.039	-0.009	-0.531	-0.098	0.056	0.596
	PC5	-0.036	0.039	-0.015	-0.911	-0.113	0.041	0.362
	PC6	-0.094	0.038	-0.041	-2.440	-0.169	-0.018	0.015
	prsAnxiety * adversity	0.000	0.000	-0.037	-0.183	0.000	0.000	0.854

Model	R	R^2	Adjusted R^2	RMSE	R^2 Change	df1	df2	p
H_0	0.222	0.049	0.046	0.028	0.049	11	3377	0.000
H_1	0.222	0.049	0.046	0.028	0.000	1	3376	0.854

Notes. H_1 model includes interaction between PRS Anxiety and adversity.

Table S35. Association between functional connectome modularity and PRS Anxiety, and its interaction with adversity

Model		Coef	Std Err	Std Coef	t	Lower 95% CI	Upper 95% CI	p
H_0	*(Intercept)*	0.444	0.014		31.235	0.416	0.472	0.000
	prsAnxiety	0.000	0.000	0.017	0.983	0.000	0.001	0.326
	phenoAnxiety	0.000	0.000	0.006	0.347	0.000	0.001	0.728
	adversity	0.000	0.000	0.003	0.168	-0.001	0.001	0.866
	sex	0.015	0.001		10.784	0.012	0.017	0.000
	PC1	0.125	0.061	0.036	2.049	0.005	0.244	0.041
	PC1	0.125	0.061	0.036	2.049	0.005	0.244	0.041
	PC2	0.044	0.061	0.012	0.713	-0.076	0.164	0.476
	PC3	-0.036	0.059	-0.010	-0.606	-0.151	0.080	0.545
	PC4	-0.079	0.056	-0.024	-1.395	-0.189	0.032	0.163
	PC5	-0.128	0.056	-0.039	-2.276	-0.239	-0.018	0.023
	PC6	-0.142	0.055	-0.044	-2.583	-0.250	-0.034	0.010
H_1	*(Intercept)*	0.451	0.016		29.068	0.420	0.481	0.000
	prsAnxiety	0.000	0.000	0.031	1.438	0.000	0.001	0.151
	phenoAnxiety	0.000	0.000	0.006	0.344	0.000	0.001	0.731
	adversity	-0.005	0.005	-0.223	-1.082	-0.014	0.004	0.279
	age	0.000	0.000	0.062	3.657	0.000	0.001	0.000
	sex	0.015	0.001		10.784	0.012	0.017	0.000
	PC1	0.123	0.061	0.036	2.013	0.003	0.242	0.044
	PC2	0.044	0.061	0.012	0.722	-0.076	0.164	0.470
	PC3	-0.037	0.059	-0.011	-0.629	-0.152	0.078	0.530
	PC4	-0.080	0.056	-0.024	-1.415	-0.190	0.031	0.157
	PC5	-0.128	0.056	-0.038	-2.265	-0.238	-0.017	0.024
	PC6	-0.142	0.055	-0.044	-2.587	-0.250	-0.034	0.010
	prsAnxiety ✻ adversity	0.000	0.000	-0.226	-1.101	0.000	0.000	0.271

Model	R	R^2	Adjusted R^2	RMSE	R^2 Change	df1	df2	p
H_0	0.204	0.042	0.038	0.040	0.042	11	3377	0.000
H_1	0.205	0.042	0.039	0.040	0.000	1	3376	0.271

Notes. H_1 model includes interaction between PRS Anxiety and adversity.

Table S36. Association between functional connectome assortativity and PRS Anxiety, and its interaction with adversity

Model		Coef	Std Err	Std Coef	t	Lower 95% CI	Upper 95% CI	p
H_0	*(Intercept)*	-0.040	0.018		-2.188	-0.075	-0.004	0.029
	prsAnxiety	0.000	0.000	0.005	0.284	0.000	0.001	0.776
	phenoAnxiety	-0.001	0.000	-0.031	-1.751	-0.001	0.000	0.080
	adversity	0.002	0.001	0.061	3.376	0.001	0.003	0.001
	age	0.000	0.000	-0.032	-1.850	0.000	0.000	0.064
	sex	0.002	0.002		0.902	-0.002	0.005	0.367
	PC1	-0.393	0.078	-0.090	-5.051	-0.546	-0.240	0.000
	PC2	-0.109	0.078	-0.024	-1.389	-0.262	0.045	0.165
	PC3	-0.040	0.075	-0.009	-0.533	-0.187	0.107	0.594
	PC4	0.018	0.072	0.004	0.252	-0.123	0.159	0.801
	PC5	0.225	0.072	0.053	3.117	0.083	0.366	0.002
	PC6	0.203	0.070	0.049	2.879	0.065	0.341	0.004
H_1	*(Intercept)*	-0.040	0.020		-2.032	-0.079	-0.001	0.042
	prsAnxiety	0.000	0.000	0.004	0.193	-0.001	0.001	0.847
	phenoAnxiety	-0.001	0.000	-0.031	-1.750	-0.001	0.000	0.080
	adversity	0.002	0.006	0.075	0.362	-0.010	0.014	0.717
H_1	age	0.000	0.000	-0.032	-1.850	0.000	0.000	0.064
	sex	0.002	0.002		0.902	-0.002	0.005	0.367
	PC1	-0.393	0.078	-0.090	-5.045	-0.546	-0.240	0.000
	PC2	-0.109	0.078	-0.024	-1.389	-0.262	0.045	0.165
	PC3	-0.040	0.075	-0.009	-0.531	-0.187	0.108	0.595
	PC4	0.018	0.072	0.004	0.253	-0.123	0.159	0.800
	PC5	0.225	0.072	0.053	3.116	0.083	0.366	0.002
	PC6	0.203	0.070	0.049	2.879	0.065	0.341	0.004
	prsAnxiety ✻ adversity	0.000	0.000	0.014	0.068	0.000	0.000	0.945

Model	R	R^2	Adjusted R^2	RMSE	R^2 Change	df1	df2	p
H_0	0.145	0.021	0.018	0.051	0.021	11	3377	0.000
H_1	0.145	0.021	0.018	0.051	0.000	1	3376	0.945

Notes. H_1 model includes interaction between PRS Anxiety and adversity.

Table S37. Association between functional connectome clustering and PRS Depression, and its interaction with adversity

Model		Coef	Std Err	Std Coef	t	Lower 95% CI	Upper 95% CI	p
H_0	*(Intercept)*	-0.029	0.020		-1.479	-0.067	0.009	0.139
	prsDepression	0.000	0.000	0.022	1.117	0.000	0.001	0.264
	phenoDepression	0.000	0.000	-0.004	-0.219	-0.001	0.001	0.827
	adversity	0.002	0.001	0.053	2.881	0.000	0.003	0.004
	age	0.000	0.000	-0.032	-1.855	0.000	0.000	0.064
	sex	0.002	0.002		0.891	-0.002	0.005	0.373
	PC1	-0.364	0.083	-0.083	-4.362	-0.527	-0.200	0.000
	PC2	-0.092	0.079	-0.020	-1.156	-0.247	0.064	0.248
	PC3	-0.042	0.075	-0.010	-0.561	-0.190	0.105	0.575
	PC4	0.009	0.072	0.002	0.121	-0.133	0.150	0.904
	PC5	0.226	0.072	0.054	3.140	0.085	0.368	0.002
	PC6	0.203	0.070	0.049	2.885	0.065	0.341	0.004
H_1	*(Intercept)*	-0.021	0.021		-1.007	-0.063	0.020	0.314
	prsDepression	0.001	0.000	0.032	1.406	0.000	0.002	0.160
	phenoDepression	0.000	0.000	-0.003	-0.173	-0.001	0.001	0.862
	adversity	-0.004	0.007	-0.142	-0.629	-0.017	0.009	0.529
	age	0.000	0.000	-0.032	-1.872	0.000	0.000	0.061
	sex	0.002	0.002		0.889	-0.002	0.005	0.374
	PC1	-0.373	0.084	-0.085	-4.436	-0.537	-0.208	0.000
	PC2	-0.088	0.079	-0.019	-1.102	-0.243	0.068	0.271
	PC3	-0.045	0.075	-0.010	-0.601	-0.193	0.102	0.548
	PC4	0.007	0.072	0.002	0.099	-0.134	0.149	0.921
	PC5	0.226	0.072	0.054	3.138	0.085	0.367	0.002
	PC6	0.204	0.070	0.050	2.904	0.066	0.343	0.004
	prsDepression ✻ adversity	0.000	0.000	-0.194	-0.867	-0.001	0.000	0.386

Model	R	R^2	Adjusted R^2	RMSE	R^2 Change	df1	df2	p
H_0	0.143	0.020	0.017	0.051	0.020	11	3377	0.000
H_1	0.144	0.021	0.017	0.051	0.000	1	3376	0.386

Notes. H_1 model includes interaction between PRS Depression and adversity.

Table S38. Association between functional connectome modularity and PRS Depression, and its interaction with adversity

Model		Coef	Std Err	Std Coef	t	Lower 95% CI	Upper 95% CI	p
H_0	*(Intercept)*	0.432	0.015		28.264	0.402	0.462	0.000
	prsDepression	0.000	0.000	-0.007	-0.358	-0.001	0.001	0.720
	phenoDepression	0.000	0.000	-0.007	-0.398	-0.001	0.001	0.691
	adversity	0.000	0.000	0.008	0.446	-0.001	0.001	0.656
	age	0.000	0.000	0.062	3.686	0.000	0.001	0.000
	sex	0.015	0.001		10.790	0.012	0.017	0.000
	PC1	0.103	0.065	0.030	1.586	-0.024	0.231	0.113
	PC2	0.030	0.062	0.008	0.490	-0.091	0.152	0.624
	PC3	-0.033	0.059	-0.009	-0.555	-0.148	0.083	0.579
	PC4	-0.069	0.056	-0.021	-1.227	-0.180	0.041	0.220
	PC5	-0.130	0.056	-0.039	-2.315	-0.241	-0.020	0.021
	PC6	-0.143	0.055	-0.044	-2.600	-0.251	-0.035	0.009
H_1	*(Intercept)*	0.431	0.017		25.836	0.398	0.463	0.000
	prsDepression	0.000	0.000	-0.008	-0.368	-0.001	0.001	0.713
	phenoDepression	0.000	0.000	-0.007	-0.404	-0.001	0.001	0.687
	adversity	0.001	0.005	0.035	0.157	-0.009	0.011	0.875
	age	0.000	0.000	0.062	3.687	0.000	0.001	0.000
	sex	0.015	0.001		10.788	0.012	0.017	0.000
	PC1	0.104	0.066	0.030	1.589	-0.024	0.233	0.112
	PC2	0.030	0.062	0.008	0.482	-0.092	0.152	0.630
	PC3	-0.032	0.059	-0.009	-0.549	-0.148	0.083	0.583
	PC4	-0.069	0.056	-0.021	-1.223	-0.180	0.042	0.221
	PC5	-0.130	0.056	-0.039	-2.315	-0.241	-0.020	0.021
	PC6	-0.143	0.055	-0.044	-2.602	-0.251	-0.035	0.009
	prsDepression ✻ adversity	0.000	0.000	0.027	0.121	0.000	0.000	0.904

Model	R	R^2	Adjusted R^2	RMSE	R^2 Change	df1	df2	p
H_0	0.203	0.041	0.038	0.040	0.041	11	3377	0.000
H_1	0.203	0.041	0.038	0.040	0.000	1	3376	0.904

Notes. H_1 model includes interaction between PRS Depression and adversity.

Table S39. Association between functional connectome assortativity and PRS Depression, and its interaction with adversity

Model		Coef	Std Err	Std Coef	t	Lower 95% CI	Upper 95% CI	p
H_0	*(Intercept)*	0.432	0.015		28.264	0.402	0.462	0.000
	prsDepression	0.000	0.000	-0.007	-0.358	-0.001	0.001	0.720
	phenoDepression	0.000	0.000	-0.007	-0.398	-0.001	0.001	0.691
	adversity	0.000	0.000	0.008	0.446	-0.001	0.001	0.656
	age	0.000	0.000	0.062	3.686	0.000	0.001	0.000
	sex	0.015	0.001		10.790	0.012	0.017	0.000
	PC1	0.103	0.065	0.030	1.586	-0.024	0.231	0.113
	PC2	0.030	0.062	0.008	0.490	-0.091	0.152	0.624
	PC3	-0.033	0.059	-0.009	-0.555	-0.148	0.083	0.579
	PC4	-0.069	0.056	-0.021	-1.227	-0.180	0.041	0.220
	PC5	-0.130	0.056	-0.039	-2.315	-0.241	-0.020	0.021
	PC6	-0.143	0.055	-0.044	-2.600	-0.251	-0.035	0.009
H_1	*(Intercept)*	0.431	0.017		25.836	0.398	0.463	0.000
	prsDepression	0.000	0.000	-0.008	-0.368	-0.001	0.001	0.713
	phenoDepression	0.000	0.000	-0.007	-0.404	-0.001	0.001	0.687
	adversity	0.001	0.005	0.035	0.157	-0.009	0.011	0.875
	age	0.000	0.000	0.062	3.687	0.000	0.001	0.000
	sex	0.015	0.001		10.788	0.012	0.017	0.000
	PC1	0.104	0.066	0.030	1.589	-0.024	0.233	0.112
	PC2	0.030	0.062	0.008	0.482	-0.092	0.152	0.630
	PC3	-0.032	0.059	-0.009	-0.549	-0.148	0.083	0.583
	PC4	-0.069	0.056	-0.021	-1.223	-0.180	0.042	0.221
	PC5	-0.130	0.056	-0.039	-2.315	-0.241	-0.020	0.021
	PC6	-0.143	0.055	-0.044	-2.602	-0.251	-0.035	0.009
	prsDepression * adversity	0.000	0.000	0.027	0.121	0.000	0.000	0.904

Model	R	R^2	Adjusted R^2	RMSE	R^2 Change	df1	df2	p
H_0	0.203	0.041	0.038	0.040	0.041	11	3377	0.000
H_1	0.203	0.041	0.038	0.040	0.000	1	3376	0.904

Notes. H_1 model includes interaction between PRS Depression and adversity

Table S40. Association between functional connectome clustering and PRS Psychosis, and its interaction with adversity

Model		Coef	Std Err	Std Coef	t	Lower 95% CI	Upper 95% CI	p
H_0	*(Intercept)*	0.223	0.009		24.258	0.205	0.241	0.000
	prsPsychosis	0.000	0.000	0.001	0.067	0.000	0.000	0.947
	phenoPsychosis	0.000	0.000	-0.070	-4.106	0.000	0.000	0.000
	adversity	0.000	0.000	-0.008	-0.471	-0.001	0.000	0.638
	age	0.000	0.000	0.086	5.124	0.000	0.000	0.000
	sex	0.009	0.001		9.097	0.007	0.011	0.000
	PC1	0.277	0.042	0.114	6.666	0.196	0.359	0.000
	PC2	0.013	0.042	0.005	0.295	-0.071	0.096	0.768
	PC3	0.017	0.041	0.007	0.406	-0.064	0.097	0.685
	PC4	-0.011	0.039	-0.005	-0.280	-0.088	0.066	0.779
	PC5	-0.036	0.039	-0.015	-0.912	-0.113	0.041	0.362
	PC6	-0.091	0.038	-0.040	-2.367	-0.166	-0.016	0.018
H_1	*(Intercept)*	0.220	0.010		22.221	0.201	0.240	0.000
	prsPsychosis	0.000	0.000	-0.009	-0.411	-0.001	0.000	0.681
	phenoPsychosis	0.000	0.000	-0.069	-4.057	0.000	0.000	0.000
	adversity	0.002	0.003	0.123	0.719	-0.003	0.007	0.472
	age	0.000	0.000	0.086	5.121	0.000	0.000	0.000
	sex	0.009	0.001		9.092	0.007	0.011	0.000
	PC1	0.279	0.042	0.115	6.696	0.197	0.361	0.000
	PC2	0.011	0.043	0.004	0.251	-0.073	0.094	0.801
	PC3	0.016	0.041	0.007	0.391	-0.064	0.096	0.696
	PC4	-0.011	0.039	-0.005	-0.288	-0.088	0.066	0.773
	PC5	-0.035	0.039	-0.015	-0.897	-0.113	0.042	0.370
	PC6	-0.090	0.038	-0.039	-2.353	-0.166	-0.015	0.019
	prsPsychosis ✻ adversity	0.000	0.000	0.131	0.771	0.000	0.000	0.441

Model	R	R^2	Adjusted R^2	RMSE	R^2 Change	df1	df2	p
H_0	0.232	0.054	0.051	0.028	0.054	11	3378	0.000
H_1	0.232	0.054	0.051	0.028	0.000	1	3377	0.441

Notes. H_1 model includes interaction between PRS Psychosis and adversity

Table S41. Association between functional connectome modularity and PRS Psychosis, and its interaction with adversity

		Coef	Std Err	Std Coef	t	Lower 95% CI	Upper 95% CI	p
H_0	*(Intercept)*	0.429	0.013		32.614	0.404	0.455	0.000
	prsPsychosis	0.000	0.000	-0.023	-1.336	-0.001	0.000	0.182
	phenoPsychosis	0.000	0.000	-0.053	-3.093	0.000	0.000	0.002
	adversity	0.000	0.000	0.013	0.732	0.000	0.001	0.464
	age	0.000	0.000	0.058	3.459	0.000	0.001	0.001
	sex	0.015	0.001		10.765	0.012	0.017	0.000
	PC1	0.094	0.060	0.027	1.585	-0.022	0.211	0.113
	PC2	0.032	0.061	0.009	0.521	-0.087	0.151	0.603
	PC3	-0.044	0.059	-0.013	-0.743	-0.159	0.071	0.458
	PC4	-0.075	0.056	-0.023	-1.344	-0.185	0.035	0.179
	PC5	-0.122	0.056	-0.037	-2.154	-0.232	-0.011	0.031
	PC6	-0.138	0.055	-0.042	-2.510	-0.246	-0.030	0.012
H_1	*(Intercept)*	0.427	0.014		30.102	0.399	0.455	0.000
	prsPsychosis	-0.001	0.000	-0.028	-1.323	-0.001	0.000	0.186
	phenoPsychosis	0.000	0.000	-0.053	-3.065	0.000	0.000	0.002
	adversity	0.002	0.004	0.085	0.499	-0.006	0.010	0.618
	age	0.000	0.000	0.058	3.457	0.000	0.001	0.001
	sex	0.015	0.001		10.762	0.012	0.017	0.000
	PC1	0.096	0.060	0.028	1.604	-0.021	0.213	0.109
	PC2	0.030	0.061	0.008	0.496	-0.089	0.149	0.620
	PC3	-0.044	0.059	-0.013	-0.751	-0.159	0.071	0.453
	PC4	-0.076	0.056	-0.023	-1.348	-0.186	0.034	0.178
	PC5	-0.121	0.056	-0.036	-2.145	-0.232	-0.010	0.032
	PC6	-0.137	0.055	-0.042	-2.502	-0.245	-0.030	0.012
	prsPsychosis ✻ adversity	0.000	0.000	0.073	0.426	0.000	0.000	0.670

Model	R	R^2	Adjusted R^2	RMSE	R^2 Change	df1	df2	p
H_0	0.211	0.045	0.041	0.040	0.045	11	3378	0.000
H_1	0.211	0.045	0.041	0.040	0.000	1	3377	0.670

Notes. H_1 model includes interaction between PRS Psychosis and adversity

Table S42. Association between functional connectome assortativity and PRS Psychosis, and its interaction with adversity

Model		Coef	Std Err	Std Coef	t	Lower 95% CI	Upper 95% CI	p
H_0	*(Intercept)*	-0.049	0.017		-2.901	-0.082	-0.016	0.004
	prsPsychosis	0.000	0.000	-0.001	-0.065	-0.001	0.001	0.948
	phenoPsychosis	0.000	0.000	0.056	3.207	0.000	0.000	0.001
	adversity	0.001	0.001	0.046	2.630	0.000	0.002	0.009
	age	0.000	0.000	-0.028	-1.640	0.000	0.000	0.101
	sex	0.002	0.002		0.955	-0.002	0.005	0.340
	PC1	-0.385	0.076	-0.088	-5.056	-0.535	-0.236	0.000
	PC2	-0.097	0.078	-0.021	-1.245	-0.249	0.056	0.213
	PC3	-0.028	0.075	-0.006	-0.375	-0.176	0.119	0.707
	PC4	0.014	0.072	0.003	0.200	-0.126	0.155	0.841
	PC5	0.223	0.072	0.053	3.091	0.082	0.365	0.002
	PC6	0.204	0.070	0.049	2.899	0.066	0.342	0.004
H_1	*(Intercept)*	-0.040	0.018		-2.176	-0.075	-0.004	0.030
	prsPsychosis	0.000	0.000	0.017	0.781	-0.001	0.001	0.435
	phenoPsychosis	0.000	0.000	0.054	3.127	0.000	0.000	0.002
	adversity	-0.006	0.005	-0.192	-1.110	-0.015	0.004	0.267
	age	0.000	0.000	-0.028	-1.635	0.000	0.000	0.102
	sex	0.002	0.002		0.962	-0.002	0.005	0.336
	PC1	-0.391	0.076	-0.089	-5.120	-0.540	-0.241	0.000
	PC2	-0.091	0.078	-0.020	-1.166	-0.243	0.062	0.244
	PC3	-0.026	0.075	-0.006	-0.348	-0.173	0.121	0.728
	PC4	0.015	0.072	0.004	0.215	-0.125	0.156	0.830
	PC5	0.221	0.072	0.053	3.064	0.080	0.363	0.002
	PC6	0.202	0.070	0.049	2.875	0.064	0.340	0.004
	prsPsychosis ✻ adversity	0.000	0.000	-0.240	-1.385	-0.001	0.000	0.166

Model	R	R^2	Adjusted R^2	RMSE	R^2 Change	df1	df2	p
H_0	0.152	0.023	0.020	0.051	0.023	11	3378	0.000
H_1	0.154	0.024	0.020	0.051	0.001	1	3377	0.166

Notes. H_1 model includes interaction between PRS Psychosis and adversity

Table S43. Comparison of main and interaction effects across adversity types in relation to prsCVgeneral

**Predictor**	**β (Main Effect)**	**β* (Main Std)**	**p**	**β (Interaction)**	**β* (Int Std)**	**p**
Cumulative Adversity	0.130	0.243	**<.001**	-0.005	-0.013	0.400
Household/Community Instability	**0.190**	**0.148**	**<.001**	0.002	0.001	0.924
Physical/Sexual Abuse	0.052	0.019	0.110	0.038	0.014	0.254
Parental Neglect	**0.262**	**0.175**	**<.001**	-0.017	-0.048	0.353
Financial Difficulties	**0.114**	**0.114**	**<.001**	-0.015	-0.016	0.243

Notes. Adjusted for age, sex, and 6 PCs

Table S44. Comparison of main and interaction effects across adversity types in relation to prsCVneurodev

**Predictor**	**β (Main Effect)**	**β* (Main Std)**	**p**	**β (Interaction)**	**β* (Int Std)**	**p**
Cumulative Adversity	**-0.051**	**-0.096**	**<.001**	**0.019**	**0.043**	**<.001**
Household/Community Instability	**-0.044**	**-0.034**	**0.010**	**0.053**	**0.050**	**0.002**
Physical/Sexual Abuse	-0.031	-0.011	0.370	0.032	0.010	0.428
Parental Neglect	-0.036	-0.024	0.056	0.017	0.048	0.376
Financial Difficulties	**-0.064**	**-0.064**	**<.001**	-0.006	-0.006	0.677

Notes. Adjusted for age, sex, and 6 PCs
